# Low frequency variants can predetermine antiviral drug resistance development in herpes simplex virus type 1

**DOI:** 10.1371/journal.ppat.1014296

**Published:** 2026-06-08

**Authors:** Lena Jaki, Florian Full, Udo Gieraths, Valeria Falcone, Zsolt Ruzsics, Hartmut Hengel, Marcus Panning, Jonas Fuchs

**Affiliations:** 1 Faculty of Medicine, Institute of Virology, Freiburg University Medical Center, University of Freiburg, Freiburg, Germany; 2 Section Clinical Virus Genomics, Institute of Virology, University Medical Center Freiburg, Freiburg, Germany; 3 German Consulting Laboratory for HSV and VZV, and Medical Center— Faculty of Medicine‌‌, University of Freiburg, Freiburg, Germany; Washington State University, UNITED STATES OF AMERICA

## Abstract

Severe HSV-1 disease is treated with potent antiviral drugs, in particular aciclovir (ACV) and its derivatives. However, long-term drug exposure in immunocompromised patients can lead to the emergence of ACV-resistant HSV-1 strains and clinical treatment failure. To understand how phenotypic resistances develop on a genomic level, we analyzed the influence of ACV selection pressure on the viral genome of different HSV-1 virus strains *in vitro*. Growth kinetics and IC_50_ determination showed ACV resistance development within a single passage. Next, we performed ultra-deep, non-targeted full-genome Illumina sequencing of the parental and ACV-adapted HSV-1 strains. Interestingly, resistance-conferring mutations rapidly arose in the viral genes *UL23* and *UL30* and were already present in the parental ACV-naïve strains at extremely low variant frequencies. Based on these findings, we hypothesized that low- frequency mutations develop during continued viral replication. To test this hypothesis, a primary rescued recombinant K17 + strain was repeatedly passaged. Continued passaging indeed increased the proportion of a subset of minor variants and allowed resistance development after, but not before, 10 consecutive passages. In summary, we show that minor variants can facilitate adaptation of HSV-1 populations to selective pressures such as pharmacological inhibition of replication. These findings highlight that deep sequencing might allow early detection of resistance mutations potentially supporting antiviral drug stewardship.

## Author summary

Primary infections and reactivations with herpes simplex virus 1 (HSV-1) can cause life-threatening disease such as viral hepatitis or pneumonia in immunocompromised patients. Systemic aciclovir treatment is the first line drug for severe HSV-1 infection. Prolonged antiviral treatment in these high-risk groups to contain the infection is often accompanied by antiviral resistance. Despite causing a significant health burden, it is poorly understood how resistance mutations develop during drug selection pressure. By utilizing ultra-deep full genome sequencing of different virus populations, we show that preexisting mutant swarms are maintained at low frequencies in the viral population. Despite the extremely low abundance of these minor variants, their maintenance is pivotal for resistance development. We show that upon drug selection pressure resistant viral subpopulations can rapidly expand rendering the virus resistant. Monitoring low-frequency mutations during HSV-1 infection might allow early detection of resistance development.

## Introduction

Herpes simplex virus 1 (HSV-1), a member of the *Alphaherpesvirinae* subfamily, has a high seroprevalence of >60% in the human population and establishes life-long persistency upon primary infection [1]. HSV-1 reactivation predominantly causes mild symptoms in immunocompetent individuals [2]. However, primary infections and reactivations can also cause life-threatening disease such as viral hepatitis or pneumonia [3]. Beside new-born children, a high-risk group for severe disease are immunocompromised patients due to more frequent HSV-1 reactivations and lack of immunological control. First line treatment drugs are the nucleoside analogue aciclovir (ACV) and its derivatives, which are characterized by moderate side effects and low cytotoxicity [4]. Despite ACV being the treatment of choice, treatment failure due to antiviral resistances occurs in up to 48% of immunocompromised patients [4–8]. Such cases require switching to the more toxic drugs foscarnet and cidofovir [4,9,10]. To ensure efficient treatment, suspected ACV-resistant viruses need to be assessed for their drug resistance status. The target genes mediating ACV resistance are well known. For its activation, ACV must first be monophosphorylated by the *UL23-*encoded viral thymidine kinase (TK) and then further phosphorylated by host cell kinases to the active triphosphate form. ACV triphosphate acts as a substrate for the viral DNA polymerase (POL) encoded by *UL30* causing chain termination [4,11]. Therefore, mutations in the genes *UL23* and *UL30* are linked to ACV resistance. HSV resistance is typically assessed by *UL23* and *UL30* sequencing or phenotypic drug susceptibility testing. The gold standard for phenotypic testing are plaque reduction assays to analyze viral growth in the presence of the antiviral [7,12]. Genotypic assessment of mutations in *UL23* and *UL30* is typically performed using Sanger sequencing, that allows a semi-quantitative analysis of mutations above 10–20% variant frequency [7,13].

Resistant HSV-1 is a significant health burden among immunocompromised patients. Understanding resistance development could help to improve patient care. Although resistance conferring target genes are known, it is not fully understood if these mutations emerge due to selection of already preexisting variants or if they develop *de novo* upon selection pressure. Moreover, it is unclear if resistance development is accompanied by additional compensatory mutations in genes other than *UL23/UL30* [14].

To address these questions, we cultivated isolate-derived, and BAC-derived HSV-1 viruses in the presence or absence of ACV and assessed their resistance through genotypic and phenotypic analysis before and after adaptation. Resistance-associated mutations developed rapidly in cell culture under both 4 µM and 62 µM ACV selection pressure in virus isolates but to a lesser extent in BAC-derived viruses. Ultra-deep whole-genome sequencing of parental viruses showed that the development of resistance can involve selection of pre-existing, low frequency mutations that emerge and persist through continued viral replication. Moreover, mutations of adapted viruses were confined to *UL23* and *UL30* and we did not detect consistent compensatory mutations outside *UL23* and *UL30*. Therefore, we propose that genomic diversity may influence the potential of HSV-1 populations to develop resistance.

## Results

### Rapid adaptation of HSV-1 to ACV within a single cell culture passage‌‌

**Fig 1:**
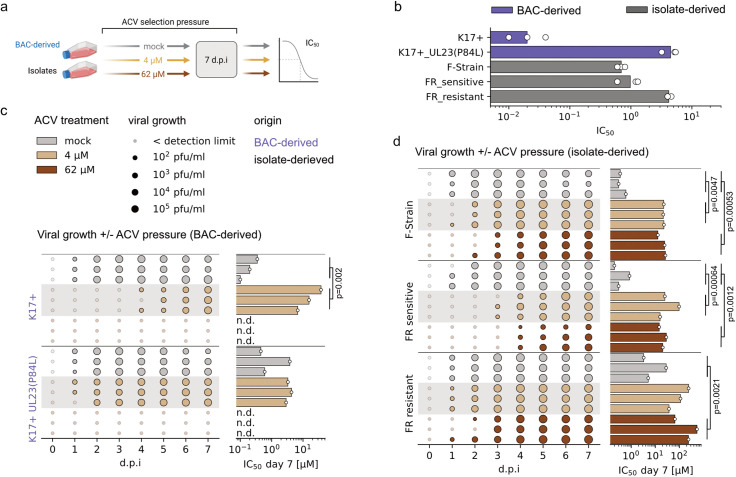
HSV-1 rapidly adapts to ACV selection pressure within a single passage. **(a)** Experimental setup of the single-passage ACV treatment regimen. BAC-derived HSV-1 or HSV-1 lab strains, originally isolated from clinical samples, were used to infect Vero cells at a moi of 0.001 (900 pfu) for 7 days in the absence (mock) or presence of 4 µM or 62 µM ACV. Every 24 h post infection, supernatant was harvested and viral growth assessed by plaque assay. Finally, ACV IC_50_ values of the supernatants harvested on day 7 were determined. Created in BioRender. Fuchs, **J.** (2026) https://BioRender.com/l5d6r7m **(b)** IC_50_ values of the parental strains prior to treatment were assessed by plaque reduction assays (geometric mean, n = 3). **(c-d)** Results of the passaging experiments described in (a) for BAC-derived viruses (c) and isolates **(d)**. Shown‌‌ is the viral growth (left plot) for each biological replicate (n = 3) where the dot size corresponds to the mean viral load of two technical replicates (pfu/ml), and the bars (right plot) the corresponding IC_50_ values of the day 7 viruses. Growth kinetics are also shown in S1 Fig. IC_50_ values of viruses grown in the absence (mock) or presence of 4 µM and 62 µM ACV were compared with a one-way ANOVA on log-transformed values. Equality of variances was confirmed with Levene’s test. Multiple comparisons with Tukey’s test were only performed for significant ANOVA results (p ≤ 0.05). P-values from multiple testing are indicated between experimental groups for p ≤ 0.05, n.d. – not determined as the viruses did not grow in the experimental setting.

To systematically analyze resistance development in HSV-1, we cultured different HSV-1 strains in the absence or presence of low dose (4 µM) or high dose (62 µM) ACV for seven days. The dosage was chosen to fall in the range of upper and lower plasma concentrations in patients treated intravenously with ACV [15] and well below reported cytotoxic concentrations in mammalian cells [16,17]. Post treatment, we harvested progeny virus and assessed its drug susceptibility by plaque reduction assays (Fig 1a‌‌). As we hypothesized that there might be differences in the phenotypic behavior of different HSV-1 strains, we chose to compare two K17 + bacterial artificial chromosomes (BAC)- derived and three isolate-derived viruses. Notably, the BAC-derived viruses were not direct rescues but already had a passaging history in order to increase their viral yield [18]. Each group included resistant viruses as a control: The ACV-resistant strain K17 + UL23(P84L), which was previously verified to be ACV resistant due to its P84L *UL23* mutation [18], and the in-house FR-ACV-resistant strain, characterized by the resistance mutation V715G in *UL30*. Indeed, IC_50_ measurements of the parental virus stocks confirmed that the two control strains were less sensitive to ACV compared to the wild type K17 + , the F-strain isolate or our in-house “FR sensitive” strain (Fig 1b). Next, we cultured the different viral strains in the absence or presence of 4 µM or 62 µM ACV for seven days (Fig 1c-1d). With the exception of the 62 µM ACV-treated, BAC-derived K17 + strain, all viruses were able to replicate in the presence or absence of ACV. While their growth followed expansion characteristics (S1 Fig), viral progenies in ACV-treated cultures were first detected with a 2–4 days delay compared to mock treated samples, but reached comparable titers to the untreated control within the following 48 h (Fig 1c-1d). Notably, the viral growth of the mock treated samples did not further increase as it was limited by the high cytopathic effect during HSV-1 infection. Interestingly, the time delay was more pronounced for the 62 µM ACV-treated viruses indicating a prolonged adaptation process during treatment with high ACV concentration. Indeed, IC_50_ values of the progeny viruses at day 7 post infection were significantly higher compared to the mock-treated control but did not significantly differ between the 4 µM and 62 µM ACV treatment.

**Fig 2:**
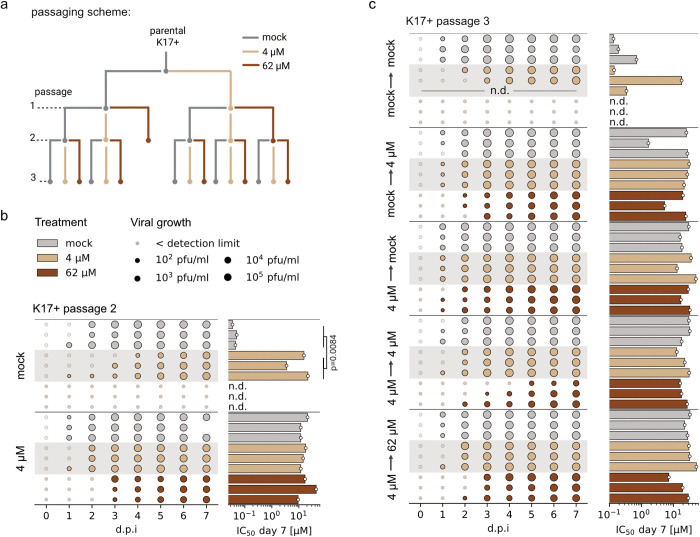
ACV resistance phenotype remains unchanged after continued passaging of the HSV-1 strain K17 + . **(a)** Passaging overview for three consecutive passages of the HSV-1 strain K17 + . Viruses from passage 1 (Fig 1) were subjected to a second and third round of passaging at an moi of 0.001 (900 pfu) in the absence (mock) or presence of 4 µM or 62 µM ACV. Every 24 h post infection, supernatant was harvested and viral growth monitored by plaque assay. Finally, ACV IC_50_ values of the supernatants harvested at day 7 were determined. **(b-c)** Results of the passaging experiments described in (a) for K17 + passage 2 (b) and for K17 + passage 3 **(c)**. Shown is the viral growth (left plot) for each biological replicate (n = 3) where the dot size corresponds to the mean viral load of two technical replicates (pfu/ml), and the bars (right plot) the corresponding IC_50_ values of the day 7 viruses. The drug treatment history is depicted on the left side of each row. Growth kinetics are also shown in S2 Fig. IC_50_ values of viruses grown in the absence (mock) or presence of 4 µM and 62 µM ACV were compared with a one-way ANOVA on log-transformed values. Equality of variances was confirmed with Levene’s test. Multiple comparisons with Tukey’s test were only performed for significant ANOVA results (p ≤ 0.05). P-values from multiple testing are indicated between experimental groups for p ≤ 0.05, n.d. – not determined as the viruses did not grow in the experimental setting.

Next, we sought to analyze the stability of the observed resistance phenotypes and to determine whether the IC_50_ values of the progeny viruses could be further increased by additional passages. To this end, the sensitive strain K17 + was passaged two additional times in the absence or presence of 4 µM or 62 µM ACV for seven days (Fig 2a). This resulted in the emergence of 5 and 15 separate adaptation lineages for passage 2 and 3, respectively (Fig 2b). Importantly, each of these lineages had different drug treatment histories (Fig 2b and 2c; left side of each row). In the second passage, we observed that the K17 + mimicked passage 1 kinetics, if previously mock treated (Figs 2b, S2). However, the 4 µM treated passage 1 was now able to replicate even in the presence of 62 µM ACV, and no further increase of IC_50_ values was recorded. A similar phenotype was observed for the third passage, as all viruses that had a drug treatment passage history now replicated under ACV treatment. IC_50_ measurements confirmed that additional passages in the presence of ACV did not further decrease ACV sensitivity and, omitting ACV after ACV treatment in a previous passage, did not restore the initial parental ACV sensitivity (Figs 2c, S2).

In summary, we showed that resistance of HSV-1 to ACV can be rapidly acquired in cell culture adapted strains within a single cell culture passage, even in the presence of high ACV doses. Moreover, the newly occurred resistance phenotypes were stable and IC_50_ values of progeny viruses could not be further enhanced by additional consecutive passages.

### ACV selection pressure coincides with mutations in UL23 and UL30 during cell culture passaging

As we observed phenotypic differences in the ACV sensitivity between BAC-derived and isolate-derived viruses, we next investigated their initial genomic diversity. We first performed ultra-deep paired end, full genome Illumina sequencing and *de novo* assembly of the parental non-adapted strains to establish highly covered consensus genomes as high-quality reference sequences were not accessible for all strains in public databases. We were able to reconstruct highly covered viral genomes with mean coverage of up to 100,000-fold (S3 Fig). Moreover, we determined the genomic makeup at very low variant frequencies by using the ultra-sensitive variant caller lofreq [19]. Throughout the analyses, we intentionally did not rely on strict variant frequency threshold, as we wanted to assess if mutations selected under evolutionary pressure by ACV were already preexisting in the original parental strain even at very low frequencies and are enriched during the selection process or if they solely develop *de novo* during ACV treatment. We identified parental low-frequency mutations in our newly constructed genomes and analyzed the number of mutations per gene and kilobase.

Low-variant frequency mutations were evenly distributed across all genes with no particular gene having more mutations across all different parental viruses indicating the steady state of a mutational, non-directed background variability (S4 Fig). Notably, differences in the number of detected variants were strain specific and likely correlated with the sequencing depth. Moreover, similar to all coding genes, the coverages of the ACV-resistance associated genes *UL23* and *UL30* showed high homogeneous per-base coverages (S4 Fig).

**Fig 3:**
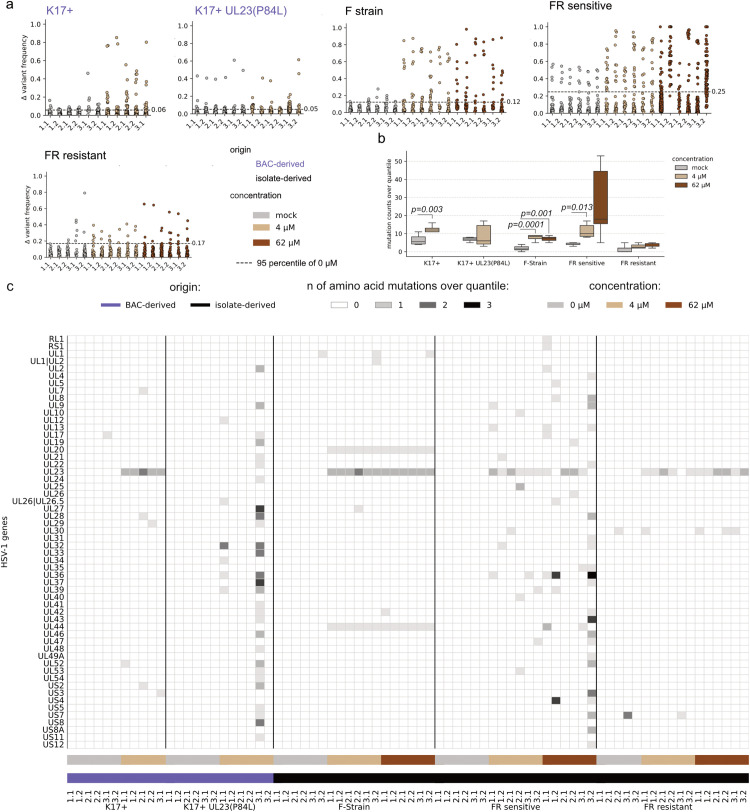
Deep Illumina sequencing of viral progeny after ACV treatment. Each technical replicate was deep-sequenced separately and mapped to its respective reference genome. Variants were then called with lofreq and annotated with BAMdash. **(a)** Dot plots showing the Δ variant frequencies of detected mutations in BAC-derived viruses and isolates. Δ variant frequencies are the absolute difference between the variant frequency of a mutation in the parental virus and the treated virus. Non-detected variants in the parental viruses were treated as having a variant frequency of 0. The dotted horizontal lines show the individual 95 percentile of a log-normal distribution fitted to the cumulative variant frequencies detected in the mock setting for each virus (see S6 Fig). X-Axis labeling indicates the biological replicate (first number) and technical replicate (second number). **(b)** Box plots of all mutations over the percentile cutoff for each treatment regimen and virus. Boxes show the upper and lower quantile and the black line indicates the median. Statistics were computed with a one-way ANOVA or Welch’s ANOVA (FR sensitive and K17 + UL23(P84L)), due to unequal variances as assessed with Levene’s test. Tukey’s or Games-Howell post-hoc multi-comparison tests were performed for significant one-way or Welch’s ANOVA results, respectively. P values are indicated compared to mock (p ≤ 0.05). **(c)** Heat map showing the number of mutations per gene present in a higher variant frequency than the percentile cutoff and conferring amino acid effects. Each column represents the sequencing results of a single replicate.

Next, we performed deep Illumina whole genome sequencing of all day 7 passage 1 HSV-1 viruses. As we hypothesized that the genomic adaptation processes could be random and sequences might therefore differ between technical replicates, we sequenced each technical duplicate of each biological triplicate separately, yielding a total of six data sets per viral strain and treatment (S1 Table). Similar to the parental virus preparations, we assessed the mutational makeup by mapping the viral reads to the parental consensus genomes. We again did not rely on a strict variant calling cutoff, as we propose that for each detected mutation the increase or decrease in its variant frequency is more important than it reaching a particular frequency. Following this rationale, for each mutation that is found in the treated viruses as well as in the respective parental strain, we computed their absolute difference in variant frequency (∆variant frequency). If the mutation is not found in the parental strain a variant frequency of zero is assumed for the parental mutation, rendering the ∆variant frequency to be equal to the variant frequency of the treated strain. Next, we determined which mutations changed in their frequency during ACV treatment more than they would in the absence of ACV, independent if they are located in the known resistance targets *UL23* and *UL30* (rationale graphically explained in S5 Fig). To this end, we model the distribution of ∆variant frequencies for mock-treated viruses, which allows us to identify a ∆variant frequency cutoff, above which changes are highly unlikely to appear in the absence of ACV treatment pressure. As we did not know which distribution best explains observed ∆variant frequencies, we first fitted probability density functions of different potential distributions to the cumulative ∆variant frequencies of all mock-treated replicates (S6a Fig). We found that the distribution of ∆variant frequencies can be best described by a log-normal distribution as shown by log-likelihood and Kolmogorov-Smirnov statistics (S6b Fig). Hence, most variant frequencies changed only marginally during mock treatment (S6a Fig). Next, we hypothesized that mutations with a statistically higher ∆variant frequency than expected from the modeled background distribution are likely to be the result of an adaptation process. Consequently, we calculated the 95^th^ percentile for each fitted distribution and used it as a ∆variant frequency cutoff for the passage 1 replicates (Fig 3a). Interestingly, the 95^th^ percentile cutoffs were highly dependent on the HSV-1 strain likely due to different diversifications in the parental virus stocks. For example, a ∆variant frequency of 0.25 in the FR sensitive strain, effectively results in a required minimum variant frequency of 25% of a mutation that is not detected in the parental strain. If for example a mutation is present in 5% in the parental strain, it has to have a variant frequency of at least 30% in the treated setting to reach the calculated ∆variant frequency cutoff. When analyzing the number of mutations with ∆variant frequencies above the established cutoffs, we found a significantly increased number of mutations in ACV-treated sensitive viruses compared to untreated viruses (Fig 3b), indicating that culturing viruses in the presence of ACV led to an overall increase in the number of higher frequency mutations.

Next, we systematically counted amino-acid change conferring mutations above the cutoff for each viral open reading frame and replicate (Fig 3c). HSV-1 strains that had reduced ACV sensitivity after drug treatment consistently showed mutations above the respective variant frequency cutoffs in the *UL23* gene (Fig 3c). Notably, this was not the case for the ACV- resistant K17 + _UL23(P84L) strain, which did not show higher IC_50_ values after ACV treatment (Fig 1b)*.* For the ACV resistant FR isolate that presents a *UL30* resistance-conferring mutation in the parental strain we observed additional mutations in both *UL23* and *UL30* upon ACV selection pressure. This coincided with an even more pronounced loss in ACV sensitivity after drug treatment (Fig 1c). Moreover, we also observed amino-acid change conferring mutations above our frequency cutoff in other genes (Fig 3c). However, these mutations occurred in various genes for various viruses and treatments. This suggests that these mutations are probably random events or adaptations to cell culture rather than compensatory mutations relevant for ACV resistance.

In conclusion, our deep-genotypic assessment prior and post ACV adaptation showed that mutations selected upon drug treatment pressure are constraint to *UL23* and to a lesser degree to *UL30*.

### UL23 and UL30 resistance-conferring mutations are selected from a large pool of pre-existing minority variants in the HSV-1 parental strains

**Fig 4:**
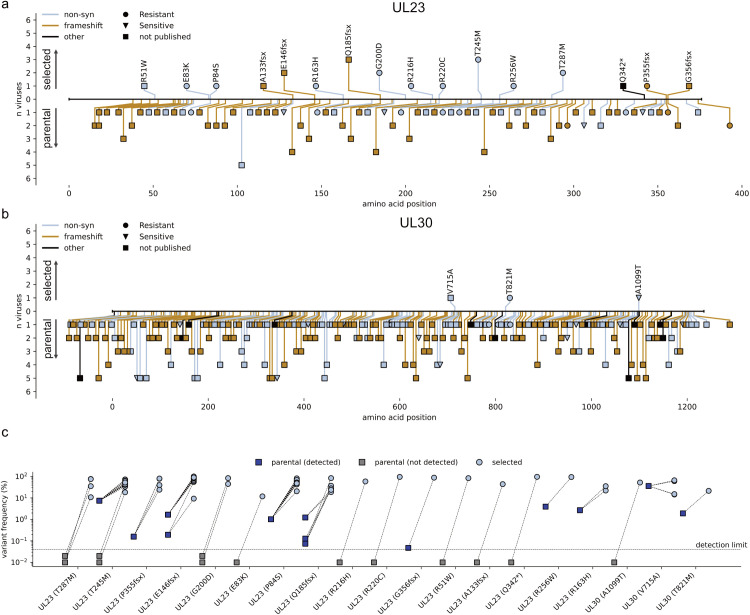
Resistance conferring mutations in *UL23* and *UL30* are already present in the parental virus stocks. Resistance was determined based on previously published data (https://doi.org/10.5281/zenodo.14351987). **(a-b)** Mutational map of amino acid change conferring mutations in **(a)** *UL23* and **(b)** *UL30* and the number of unique viruses harboring the mutation. On the positive y-axis, mutations detected after ACV treatment and exceeding the individual percentile cutoffs are shown. On the negative y-axis, mutations in the parental viruses independently of their variant frequency are depicted. The mutations are colorized by their mutational effect (blue – non-synonymous single mutations, orange – insertions or deletions resulting in a frameshift, black – mutations leading to amino acid effects that are not frameshifts or non-synonymous mutations). The dot shape depicts the result of a database comparison with previously published literature (circle – published as ACV resistant conferring, triangle – published as non-ACV resistant conferring, square – not described). **(c)** For the individual *UL23* and *UL30* amino acid conferring mutations, the variant frequencies of the parental and the variant frequencies of replicates after ACV treatment are plotted as linked dot plots. Non-detected variants in the parental viruses were set to values below the variant calling cutoff.

Next, we asked whether mutations with increased variant frequencies in *UL23* and *UL30* are known to be resistance conferring and if mutations were unique or common for different viral strains. Therefore, we calculated unique amino acid change conferring mutations in either the *UL23* or *UL30* over the ∆variant frequency cutoff in the different viruses by pooling data from all passage 1 replicates of one virus (S1 Data). Intriguingly, we identified multiple mutations in *UL23* that had been selected in more than one virus indicating convergent selection (see Fig 4a, selected). By comparing the detected mutations with a database of already published mutations [20], we confirmed that all non-synonymous mutations were associated with a decreased sensitivity to ACV. The remaining non-published mutations were frameshifts that likely render the viral TK non-functional thereby conferring ACV resistance [20]. For UL30, we detected only three unique mutations, each observed in just one replicate of either the FR-sensitive or FR-resistant strains (Fig 4b selected, Fig 3c).

Next, we analyzed the background genomic variability of parental strains without a variant frequency cutoff for both *UL23* and *UL30* (Fig 4a and 4b parental). Interestingly, we found numerous mutations in both *UL23* and *UL30* that were present in multiple parental strains, with some mutations present in all strains. We wondered whether the observed mutations in the parental strains had occurred in regions that might cause problems during viral replication (S7a Fig). This prompted us to perform sequence analyses of the sites at which insertions, deletions or single nucleotide polymorphisms (SNPs) were detected and compared our findings to 500 randomly sampled nucleotide positions over each viral genome. We assessed whether mutations were part of homopolymer stretches (S7b and S7c Fig), repeating regions (S7d-S7f Fig), regions with a higher GC content (S7g Fig) or regions with a higher Gibb’s free energy (∆G) indicating the presence of higher ordered, secondary DNA structures (S7h Fig). While repeating regions played a subsidiary role, we found that both insertions and deletions were significantly more frequent in GC-rich regions and homopolymer stretches, with a mean length of repeating nucleotides that was higher than expected at random (S7b and S7g Fig). Moreover, SNPs were overrepresented in regions with flanking homopolymer regions or regions (S7c Fig) with a lower ∆G compared to the random sample (S7h Fig). Both homopolymer stretches and secondary structures are known to impede replication by polymerase slippage and steric hindrance of the replication complex, respectively [21,22]. We hypothesize that the large variability of low frequency mutations in the parental strains develop during continued viral replication and are maintained to enable rapid adaptions to evolutionary pressures similar to mechanisms known from RNA viruses [23]. Therefore, we analyzed if the detected *UL23* and *UL30* mutations were already present in the parental viruses before selection by ACV treatment. Indeed, we found that more than half of the mutations detected were already present in the parental strains, with their variant frequency increasing up to 1000-fold upon ACV selection pressure (Fig 4c, S2 Table).

### Genome diversity determines the HSV-1 ACV resistance potential

**Fig 5:**
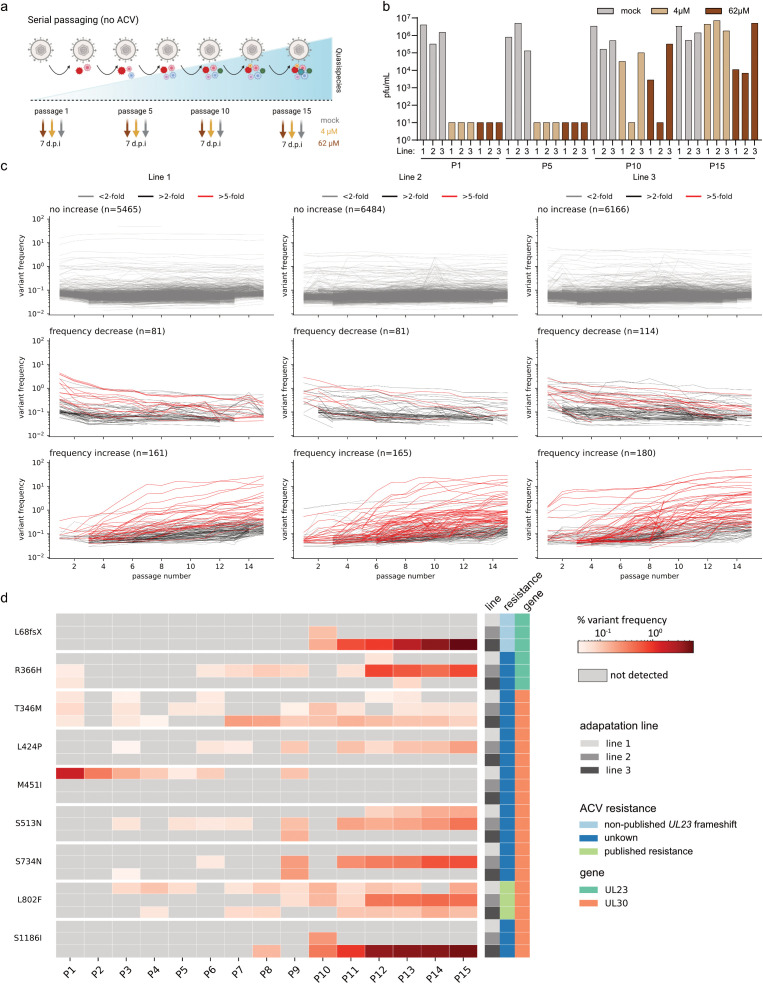
Increasing minority variants can predetermine resistance development. **(a)** Schematic representation of the experimental evolution setup using serial passaging of a primary rescued, plaque purified virus in the absence of ACV to diversify the viral population. To test if more diverse populations predetermine resistance development, progeny viruses from passage 1, 5, 10 and 15 were further cultured in the presence/absence of ACV for seven days. Created in BioRender. Fuchs, **J.** (2026) https://BioRender.com/7dzqsfa **(b)** BAC-derived K17 + was rescued in Vero cells and plaque purified. The individual plaques were cultured for 48 **h.** Next, every 48 h, 10 µl supernatant was transferred to fresh Vero cells for a total of 15 passages (n = 3). Supernatants of passage 1, 5, 10 and 15 were used to infect Vero cells (moi = 0.001) in the absence (mock) or presence of 4 µM or 62 µM ACV for 7 days. Shown are the viral titers at day 7 for each individual passage. **(c-d)** Passages were individually sequenced and variants called compared to the K17 + reference genome. Next, variants were tracked over passages and individual adaptation lines and common mutations, defined as mutations present in all lines and in one line in at least 13 passages, were excluded if they did not increase in their frequency in any line. **(c)** Variant frequencies of all mutations were tracked over the different passages. Individual mutations were separated by lines (left, middle, right) and if the passages show less than two-fold (upper plots), more than a two-fold decrease (middle plots) or more than two-fold increase in variant frequencies between the first and last detected in which they were detected**. (d)** Heatmap of variant frequencies of *UL23* and *UL30* mutations tracked over the different passages and lines. Each cell represents the variant frequency in one line and passage. Mutations were included only if their frequency changed by at least 5-fold in at least one lineage. Grey fields indicate that a mutation was not detected. Resistance was determined based on previously published data (https://doi.org/10.5281/zenodo.14351987).

Based on our previous observations, the potential to develop ACV resistance in BAC- and non-BAC-derived viruses might directly depend on the preexisting genetic diversity, e.g., due to different passaging histories. Indeed, all viruses used in our previous experiments including the BAC-derived viruses had been passaged multiple times in cell culture. We hypothesized that a primary rescued recombinant, plaque-purified virus would not be able to readily adapt to ACV selection pressure and that experimental evolution by serial passaging in the absence of ACV should increase its adaptation potential (Fig 5a). To test this assumption, we rescued BACmid derived K17 + virus and performed a plaque-purification. Three plaque-purified virus stocks were then separately passaged 15 times in the absence of ACV, creating three distinct lineages. After each passage, progeny virus was harvested 48 h p.i. and used to inoculate new cells. Viral titers reached at 48 h did not increase or decrease over the passages (S8a Fig). Remarkably, despite the absence of ACV selection pressure, we observed an increase in ACV IC_50_ values for passage 10 and 15 (S8b Fig). Next, we cultured passage 1, 5, 10 and 15 in the absence or presence of ACV and determined viral titers after 7 days. Passage 1 and 5 were not able to grow under ACV treatment, whereas two of the three lineages of passage 10 and all lineages of passage 15 replicated to high titers. IC_50_ measurements confirmed that these viruses indeed lost ACV sensitivity (S8c Fig). We performed ultra-deep sequencing of all passages prior to ACV treatment and analyzed their genetic makeup. Interestingly, we detected similar and high numbers of mutations at low variant frequencies in all passages. By tracking all 11,294 unique mutations across all separate lines and passages, we observed that a large proportion of mutations was detectable in all lines independent of the passage and are maintained during passaging from P1 onward (S8d and S8e Fig – pre filtering). Analogous to our ∆variant frequency approach, we argued that such commonly detected mutations would not be relevant for the observed phenotype if they do not change in their frequency. This prompted us to investigate how the low-frequency genotype was changing during passaging. Specifically, we define the low-frequency genotype as a genotype whose mutations either increase in their frequency or appear and disappear during passaging. Therefore, we excluded mutations that are common to all separate adaptation lines and passages. We only kept common mutations if they increased at least 5-fold in their variant frequency in one line. This led to the exclusion of 2,764 mutations showing less overlap between the independent lines (S8d Fig – post filtering). Moreover, it showed that new mutations were maintained for several passages (S8e Fig – post filtering). This indicated a fluid appearance and disappearance of mutations during passaging. The unique low-frequency genotype of each passage was therefore defined by novel mutations that appeared during this passage and mutations that originated from previous passages. However, this did not explain why passage 10 was able to confer resistance while passage 5 was not (Fig 5b). Therefore, we analyzed the variant frequency development of all mutations in each passaging line. The large majority of variants represented mutations that did not increase or decrease in their variant frequencies and was maintained below 0.1% for a couple of passages (Fig 5c upper panel). However, there were some initially low frequency mutations that indeed increased or decreased in their prevalence over several passages by up to 20-fold (Fig 5c middle and lower panel). Interestingly, there was a clear tendency of increasing variant frequencies at later passages. As the resistance potential increased with the number of passages, we hypothesized that potential resistance mutations might follow a similar trend. Therefore, we analyzed all *UL23* and *UL30* mutations that increased at least 5-fold in their frequency in at least one adaptation line (Fig 5d). Indeed, we found multiple *UL30* and *UL23* nucleotide polymorphisms that increased in their frequency over passages. Two of these mutations (L68fsX in *UL23* and L802F in *UL30*) can likely confer ACV resistance based on available literature [20].

In summary, we observed that the large majority of detected variants are maintained over several passages with only a small number of mutations increasing in their frequencies. We propose that these mutations represent the relevant mutation subset that allowed adaptations to selection pressures and might be initially selected due to other fitness advantages or by chance.

## Discussion

The clinical use of drugs targeting virus-encoded factors of the replication machinery and inhibiting the pathogenic consequences of herpes viruses is steadily increasing. However, the therapeutic success becomes limited due to the development of drug resistances which requires a better understanding of the fundamentals underlying acquisition of antiviral resistance. Consequently, the emergence and management of antiviral resistances in herpesviruses has been subject of numerous publications [24–27]. Previous studies have specifically focused on aggregating publicly available resistance conferring mutations or tracking HSV-1 evolution during resistance development [20,28,29]. However, it is still poorly understood what mechanisms drive resistance development in HSV-1 and other human herpesviruses. Our study sheds light on the role of minor variants in the emergence of ACV resistance for HSV-1. We propose that, even without selection pressure, minor variant populations expand by default through continued viral replication. We demonstrated that drug-resistance conferring low-frequency mutations can increase in their frequency by several log-folds upon ACV selection pressure. Moreover, we observed that mutations are maintained at extremely low variant frequencies below 0.1% but a small proportion increases over continued passaging.

Based on these observations, we propose that these maintained, minor variant subsets have a quasi-species like character. In contrast to RNA viruses where viral quasi-species have been extensively characterized, the concept of viral mutant swarms is not well studied for DNA viruses [23]. RNA viruses have generally higher mutations rates than DNA viruses due to the error-prone RNA-dependent RNA polymerases that typically lack an exonuclease domain for proofreading [30,31]. Error-borne diversification of an RNA virus population is one of the main evolutionary drivers allowing adaptation to evolutionary bottlenecks such as to overcome a species barrier or to evade the host’s immune response [23,32]. To date, viral quasi-species are primarily assessed by short or long read sequencing [33]. However, there are practical constraints to this approach for large DNA viruses. Due to a substantially larger genome sizes and significantly lower mutation rates [34], much more sequencing data is required to detect mutations at variant frequencies below 1%. Accordingly, we produced non-targeted, ultra-deep WGS datasets of HSV-1 virus stocks with sequencing depths of up to 100,000-fold allowing the detection of minority variants in HSV-1 populations. By including mock treated samples in our single-passage settings, we were able to separate expected mutational background variability from selection pressure associated mutations. Upon ACV treatment, a consistent mutation pattern was only observed in the expected drug targets TK and POL, encoded by the *UL23* and *UL30*, respectively. We found no indication that these mutations have to be accompanied by compensatory mutations in other genes. Notably, the intrinsic capacity of HSV-1 to adapt to ACV was independent of whether the viruses originated from BAC clones or patient isolates. The observed differences in the adaptation kinetics are likely due to differences in their regrettably unknown cell culture passage histories. Importantly, with a strictly clonal primary rescued K17 + strain, we did not observe ACV resistance development in our single passage adaptation experiment. However, continued passaging of this recombinant virus also resulted in genomic diversification of the viral population and resistance development after 10 passages. This implied that enough mutations had been acquired at high frequencies relative to the background level, allowing ACV adaptation in our experimental setting. Sequence analysis of the parental, non-adapted strains indicated that insertions and deletions primarily appeared in homopolymer stretches and SNPs in regions with lower ∆G. Therefore, we speculate that the observed mutations arose due to disruptions during viral replication by well-studied mechanisms such as polymerase slippage [22,35]. Random replication errors likely contribute to HSV-1 diversification and allows the virus to adapt to selection pressure. Notably, acquiring ACV resistance represents a relatively broad bottleneck, as the viral TK is dispensable for HSV-1 replication in cell culture and single nucleotide exchanges or even frameshifts in *UL23* are well tolerated [18]. For essential viral proteins, such as proteins of the polymerase complex, mutations are under negative selection due to their potential fitness disadvantages. This mutational tolerance is likely the main reason why the majority of resistance conferring mutations after ACV selection pressure were detected in *UL23* and not *UL30* which aligns well with previously published literature [7].

Beyond the significance of understanding virus evolution, the role of minor variants in the development of HSV-1 drug resistance has translational potential. Studies with HIV and HCV have shown that understanding viral quasi-species can directly improve patient care by tracking and predicting drug resistances [36–38]. As all conclusions of our study are derived from cell culture experiments, it is unclear if they can be seamlessly transferred to clinical settings. Schalkwijk et. al showed that for both HSV-1 and HSV-2 heterogeneous drug-resistant populations can rapidly develop in patients [39,40]. Therefore, it might be possible to assess and track the genomic sequence space of HSV-1 in infected patients and analyze the development of resistant populations by deep sequencing. Detection of low-frequency mutations may indicate whether HSV-1 has begun to deploy its potential towards developing resistance or is on the verge of becoming resistant. To show a clinical benefit of such analyses, larger cohort studies utilizing, e.g., target deep-sequencing of *UL23/UL30* should be conducted and correlated with clinical outcomes. Similar strategies have been successfully applied for RNA viruses as exemplified by combinatorial monoclonal antibody therapy for SARS-CoV-2 infected patients or highly active anti-(retro-)viral therapy for HIV and HCV patients [41–43]. Indeed, a recent study exploring combinatorial HSV-1 treatments in cell culture showed that resistance development is much more improbable [44].

Overall, this study highlights that treatment-naïve virus populations can already harbor ACV resistance conferring low frequency mutations that can be rapidly selected upon drug selection pressure. They emerge and increase in their frequency during continued viral replication diversifying the sequence space which may facilitate rapid adaptation to drug treatment. Applied to diagnostic routine, in-depth analysis of an HSV-1 population via targeted NGS could also allow for early resistance detection although the clinical relevance of such analyses have to be carefully assessed.

## Materials and methods

### Virus stock production

Virus stocks of the BACmid derived HSV1(17+)Lox-UL23(wt) and HSV1(17+)Lox-UL23(P84L) were kindly provided by Prof. Dr. Beate Sodeik [18]. All virus stocks (HSV1(17+)Lox-UL23(wt), HSV1(17+)Lox-UL23(P84L), F-Strain [45], in house FR sensitive isolate, in house FR resistant isolate) were produced by infecting adherent African green monkey kidney Vero cells at a multiplicity of infection (moi) of 0.01 (ATCC CCL-81). Therefore, Vero cells were first cultured in 1 × Dulbecco’s modified Eagle medium (DMEM) containing 5% fetal calf serum (FCS), 2 mM L-glutamine and 100 U/mL penicillin and 100 µg/mL streptomycin at 37 °C, 5% CO_2_. Afterwards, medium was replaced by virus diluted in PBS and incubated for 1.5 h at 37 °C, 5% CO_2_. Then, virus dilution was removed and medium added to the cells. Cells were incubated for 48–72 h at 37 °C, 5% CO_2_ until a cytopathic effect was visible. HSV-1 was handled under BSL-2 conditions. Cells were routinely tested for mycoplasma. All virus stocks were stored at -80 °C.

### Plaque assay

To determine viral titers, Vero cells were seeded in 12-well plates and infected with virus-dilutions from 10^-1^-10^-6^. Virus inoculum was removed and DMEM containing 0.3% agar, 2% fetal calf serum (FCS), 2 mM L-glutamine and 100 U/mL penicillin and 100 µg/mL streptomycin, 20 mM HEPES pH 7.4 and 0.1% NaHCO_3_ was added. Cells were incubated for 72 h at at 37 °C, ‌‌5% CO_2_. Afterwards cells were fixed with 3.7% formaldehyde and stained with crystal violet.

### ACV dilution, plaque reduction assay and inhibitory concentration 50 determination

Pulverized ACV (Hikma Farmaceutica, Portugal) was diluted in water to a stock concentration of 110 mM. To determine the phenotypic resistance status of the viruses before and after treatment, inhibitory concentration 50 (IC_50_) values were determined by plaque reduction assay. Therefore, Vero cells were seeded in 12-well plates and infected with 100 plaque forming units (pfu). After infection, cells were incubated for 72 h at 37 °C, ‌‌5% CO_2_ with increasing ACV concentrations ranging from 0 µM up to 2,500 µM ACV in a 1:1 mixture of infection medium and overlay (Avicel). Cells were fixed with 3.7% formaldehyde and stained with crystal violet. Plaques were counted and normalized to untreated control. To determine the IC_50_, a non-linear fit least squares regression was used (constraints: bottom constant equal to 0 and upper constant equal to 100).

### Viral growth in the presence or absence of ACV for seven days

Viral replication under drug treatment was analyzed by inoculating Vero cells in 6-well plates at an moi of 0.001 in PBS for 1.5 h at 37 °C, 5% CO_2_. Afterwards, the infection inoculum was replaced with fresh 1 × Dulbecco’s modified Eagle medium (DMEM) containing 2% fetal calf serum (FCS), 2 mM L-glutamine and 100 U/mL penicillin, 100 µg/mL streptomycin, 20 mM HEPES and 0.1% NaHCO_3_ at 37 °C, 5% CO_2_. Additionally, 4 µM or 62 µM ACV was added to the medium or left untreated (mock). Supernatant was collected at 24 h, 48 h, 72 h, 96 h, 120 h, 144 h‌‌ and 168 h post-infection. Viral titers were determined by plaque assays. Experiments were performed in technical duplicates and biological triplicates.

### Rescue of recombinant HSV-1

*Eschericha coli* carrying a BACmid containing the HSV1(17+)Lox-UL23(wt) genome were used to inoculate 200 mL LB medium with 25 µg/mL chloramphenicol and cultured over night at 37 °C. BACmid DNA was purified with NucleoBond Xtra Midi Kit für Plasmid DNA (Macherey Nagel, Düren, Germany) and transfected to Vero cells. 1 µg DNA per 6-well was mixed with Lipofectamin 3000 (Thermo Fisher, Waltham, USA) according to manufacturer’s protocol and added to Vero cells. Virus containing supernatant was harvested after culturing the cells for 72 h until CPE was visible. Plaque assay on the supernatant was performed and plaques were picked. Single plaques were used to infect Vero cells. Cells were incubated for 48‌‌ h at 37 °C‌‌ and 5% CO_2_. Afterwards, a plaque assay was performed and single plaques were transferred to new Vero cells and cultured again for 48 h to produce the final plaque purified viral stocks used in the serial passaging experiment (P0).

### Experimental evolution by serial virus passaging

For the first passage, 10 µL of a plaque purified, primary recombinant HSV1(17+)Lox-UL23(wt) rescue (passage 0) was transferred to a 6-Well of confluent Vero cells. Each line (n = 3) was infected with a different plaque purified stock (P0). Next, infected cells were incubated for 48 h at 37 °C, 5% CO_2_. Additional 14 passages were generated by transferring 10 µl of viral supernatant from the previous passage to confluent Vero cells. The calculated mean infectious dose per passage and line was 28,000 pfu (viral titers of each passage are given in S8 Fig). For each passage the viral supernatants were harvested and titers were determined to monitor the infection dose. Viral supernatants of passage 1, 5, 10 and 15 were used to perform viral growth kinetics in the presence of a mock treated, 4 µM and 62 µM ACV for seven days. However, growth was only evaluated by determining the viral titers at 168 h post infection. IC_50_ values were determined prior and post ACV selection pressure.

### Sequencing

300 µL viral supernatant were sterile filtered with PES syringe filters, diameter 13 mm, pore size 0.45 µm. DNA was purified with the Monarch Genomic DNA Purification Kit (New England Biolabs, Frankfurt am Main, Germany). 10–100 ng DNA were used for Illumina library preparation. Library preparation was done with NEBNext Ultra II FS DNA Library Prep Kit (New England Biolabs, Frankfurt am Main, Germany). Viruses were sequenced by Zymo Research Europe (Freiburg, Germany) on a NovaSeq X Plus (Illumina, San Diego, CA) platform or in house on a MiSeq (Illumina, San Diego, CA).

### Bioinformatic analysis

Raw reads generated from the parental virus stocks in this study were used to *de novo* assemble high quality references genomes with a custom Galaxy workflow available at: https://usegalaxy.eu/u/jonasfuchs/w/viral-de-novo-assembly-pipeline. Briefly, paired-end reads were preprocessed with fastp v.0.23.2 [46] and then pre-filtered for viral reads with Kraken2 [47] v.2.1.1 using the prebuild viral Refseq database (version 06.07.2022). The non-viral reads were mapped to the human genome hg19 using BWA-MEM [48] v.0.7.17. Unmapped non-human and viral reads were then used as input for the three different *de novo* assemblers Velvet 1.2.10 [49], MEGAHIT [50] v.1.2.9 and SPAdes v.3.15.4. Contigs longer than 1000 bp from all three assemblers were used to build a consensus contig using the sequence assembler cap3 [51]. Afterwards the contigs were again filtered for viral contigs with Kraken2 and all reads remapped to the newly generated contigs using BWA-MEM. A consensus sequence was generated with ivar v.1.4.2 [52] and annotated using Prokka v.1.14.6 [53]. Generated genomic sequences were manually inspected and edited using Geneious v.2019.2.3.

Paired end reads from non-parental virus stocks (growth kinetics or serial passages) were mapped to the de novo generated consensus sequence using a previously published custom Galaxy workflow [54]. Briefly, reads were pre-processed with fastp (default parameters) and mapped to the reference genome using BWA-MEM (-T ‘30’ -h ‘5’ -Y -q). Afterwards, variants were called using lofreq v.2.1.5 [19] (--call-indels --min-cov 5 --max-depth 1000000 --min-bq 30 --min-alt-bq 30 --min-mq 0 --max-mq 255 --min-jq 0 --min-alt-jq 0 --def-alt-jq 0 --sig 0.0005 --bonf dynamic --no-default-filter). To assess the full genetic makeup of the parental strains, the same workflow was used to remap the paired-end reads to the respective *de-novo* assembled reference genomes. ‌‌The workflow is available at: https://usegalaxy.eu/u/jonasfuchs/w/mapping-for-dna-viruses-with-consensus-creation-fasta-ref-1.

### Data analysis

BAMdash v.4.1 (https://github.com/jonas-fuchs/BAMdash, https://doi.org/10.5281/zenodo.8420445) was used to generate coverage plots and annotate the mutational effects of detected sequence variations on the translated amino acid coding sequence. All data were analyzed and plotted with GraphPad Prism 10 or python3.11.

## Supporting information

S1 Table Overview of the sequencing results. For each virus and treatment, the mean coverage over reference and genome recovery are shown. Genome recovery was calculated as percentage of regions covered at least 20-fold.
(PDF)

S2 Table Detected mutations in UL23/UL30 above the Δvariant frequency threshold. The numbering of each replicate corresponds to the biological replicate (first number) and technical replicate (second number). Resistance was determined based on previously published data (https://doi.org/10.5281/zenodo.14351987).
(PDF)

S1 Fig Growth kinetics of HSV-1 strains for seven days in the presence or absence of ACV. Vero cells were infected at a moi of 0.001 with the different viruses in the absence (mock) or presence of 4 µM or 62 µM ACV. Viral growth of the individual viruses was measured in biological triplicates by plaque assay. Each connected line represents one biological replicate. This Fig uses the same data as Fig 1.
(TIF)

S2 Fig Additional seven-day growth kinetics of K17+ previously cultured in the presence or absence of ACV for seven days. Vero cells were infected at a moi of 0.001 with the K17 + passaged viruses under different ACV concentrations in the absence (mock) or presence of 4 µM or 62 µM ACV. Viral growth of the individual viruses was measured in biological triplicates by plaque assay. Each connected line represents one biological replicate. The header above each Fig shows the prior passaging history. This Fig uses the same data as Fig 2.
(TIF)

S3 Fig Coverage plots of the parental viruses. Parental viruses were *de novo* assembled and reads remapped to the newly assembled genome. Coverage plots were created with BAMdash. Below the coverage, the coding sequences of the individual reference genes are plotted. The dotted lines depict the mean coverage.
(TIF)

S4 Fig Mutations in the parental viruses are not biased to specific genes. (a) Stacked bar plots showing for each parental virus the number of synonymous and non-synonymous variants/kb (left y axis) and the respective coverages per gene (right y axis). (b) Per-base coverage plots for both *UL23* and *UL30* of each parental virus.
(TIF)

S5 Fig Proposed solution on how to identify relevant mutations under selection pressure by ACV. Rationale on how to identify mutations that are likely attributed to selection during treatment. Using variant cutoffs such as 1, 5 or 10% to find such mutations can lead to edge cases where mutations in the parental virus are slightly below the variant calling cutoff and slightly above the cutoff in the treated samples. Therefore, such mutations only have a marginal increase compared to the original mutation (mutation B). Moreover, a comparison between parental and treated viruses is important independent of a variant frequency cutoff to assess if mutations actual change in their frequency (mutation A) or not (mutation C). The here proposed solution is to call variants in the absence of a variant cutoff and instead focus on relevant changes during treatment. To define relevance, Δvariant frequencies are calculated and for the mock treated condition a distribution is modelled to define the 95^th^ percentile above which mutations have a higher change in frequency than expected. We propose that in a setting with ACV treatment using this Δvariant frequency cutoff will lead to the identification of mutations that change significantly in their frequency. Created in BioRender. Fuchs, J. (2026) https://BioRender.com/6ya2i42.
(TIF)

S6 Fig Distribution fitting to the cumulative Δ variant frequencies of mutations detected in all mock treated replicates. To assess the mutational background noise expected in the absence of ACV selection pressure, the cumulative Δ variant frequencies were evaluated for each mock-treated strain separately as the observed mutations and their respective frequencies were dependent on the parental virus stocks. **(a)** Therefore, different distributions were fitted to the Δ variant frequencies of the PBS treated control: log-normal distribution (lognorm PDF), normal distribution (norm PDF), gamma distribution (gamma PDF), Weibull distribution (weibull_min PDF) and exponential distribution (expon PDF). Δ variant frequencies are the absolute difference between the variant frequency of a mutation in the parental virus and the treated virus. Non-detected variants in the parental viruses were treated as having a variant frequency of 0. PDF – probability density function. **(b)** Distribution fitting was systematically evaluated by the Kolmogorov–Smirnov test and maximum likelihood estimation. Shown are the results for each test. Bold marked are the values that indicate the best fitting distribution for each test (Kolmogorov–Smirnov statistic: higher is better, Kolmogorov–Smirnov p-value: lower is better, log-likelihood: higher is better). For all viruses log-normal distributions fitted best to the Δ variant frequencies.
(TIF)

S7 Fig Patterns at mutations site in the parental viruses. **(a)** Stacked bar plots depicting the percentage of insertions (INS), deletions (DEL) and single nucleotide variants (SNP) for each parental virus. **(b-h)** Using python3, sites of mutations were systematically evaluated for sequence patterns compared to 500 randomly selected positions in the viral reference sequence. Each dot represents the mean of all tested mutations per virus and substitution type. The horizontal lines indicate the mean. Examples for each pattern is given above the graph (underlined) with the site of mutation marked in red. (b) Percentage of mutations that were part of homopolymer stretches longer than 3 nucleotides (left) and the mean length of the detected stretch (right). (c) Percentage of mutations that had flanking homopolymer stretches longer than 3 nucleotides (left) and the mean length of the detected stretches (right). (d-f) Percentage of mutations that were part of di-nucleotide (d), tri-nucleotide (e) or quad-nucleotide repeats (f) of at least 4, 3 and 2 repeats, respectively. (g) GC content and (h) deltaG (left) of the flanking sequence region consisting of 10 nucleotides up- and downstream of the mutation’s regions were analyzed. (b-h) Equality of variances were tested with Levene’s test. Statistics were calculated with a one-way ANOVA together with Tukey multiple comparison test (c – left, f) for equal variances and Welch’s ANOVA together with Games-Howell multiple comparison test (remaining Figs). Multiple testing was only performed for significant ANOVA results (p ≤ 0.05). P-values from the multiple testing are indicated for p ≤ 0.05.
(TIF)

S8 Fig Passaging of K17+. **(a)** Viral titers of each passage after 48 h post infection with 10 µl/well of a 6-well plate of the previous passage. Connected dots represent one consecutive passage. **(b)** IC_50_ values of passage 1, 5, 10 and 15 without selection pressure. Statistics were calculated with a one-way ANOVA together with Tukey multiple comparison test. P-values from the multiple testing are indicated for p ≤ 0.05. **(c)** IC_50_ values after successful culturing the individual replicates of passage 10 and 15 in the absence (mock) or presence of 4 µM or 62 µM ACV for 7 days. **(d-e)** Passages were individually sequenced and variants called compared to the K17 + reference genome. Next, variants were tracked over passages and individual adaptation lines (pre-filtering). Common mutations, defined as mutations present in all lines and in one line in at least 13 passages, were excluded if they did not increase in their frequency in any line (post filtering). (c) Venn-diagrams showing the number of mutations overlapping pre- and post-filtering between lines. (d) Sankey plot showing in which passage a mutation first and last appeared pre- and post-filtering.
(TIF)

S1 Data‌‌ UL23 and UL30 mutations detected in the parental strains prior ACV selection and their know effect on antiviral resistance. The data given here are the basis for the parental mutations depicted in Fig 4a and 4b. The genetic makeup of the parental strains was analogously analyzed to the passaged viruses by remapping the paired-end reads back to the respective *de-novo* assembled reference genomes using the same bioinformatic workflow.‌‌
(XLSX)
