# Supplementary material for: Low frequency variants can predetermine antiviral drug resistance development in herpes simplex virus type 1
Source: PLoS Pathog. 2026 Jun 8;22(6):e1014296. doi: 10.1371/journal.ppat.1014296 (PMC13262928; doi:10.1371/journal.ppat.1014296)
Supplement: S1 Table — For each virus and treatment, the mean coverage over reference and genome recovery are shown. Genome recovery was calculated as percentage of regions covered at least 20-fold. (PDF) [file ppat.1014296.s001.pdf]

Virus	Concentration of treatment	Biological replicate	Technical replicate	Mean coverage	% genome recovery
**K17+**	0	1	1	471	97
	0	1	2	541	97
	0	2	1	515	97
	0	2	2	855	97
	0	3	1	63	96
	0	3	2	1328	97
	4	1	1	3836	97
	4	1	2	663	97
	4	2	1	1186	97
	4	2	2	n.s.	n.s.
	4	3	1	5496	97
	4	3	2	n.s.	n.s.
	62	1	1	n.s.	n.s.
	62	1	2	n.s.	n.s.
	62	2	1	n.s.	n.s.
	62	2	2	n.s.	n.s.
	62	3	1	n.s.	n.s.
	62	3	2	n.s.	n.s.
	Parental			33203	98
**K17+_UL23(P84L)**	0	1	1	459	97
	0	1	2	733	97
	0	2	1	652	97
	0	2	2	865	97
	0	3	1	181	97
	0	3	2	2382	97
	4	1	1	50	97
	4	1	2	171	97
	4	2	1	116	97
	4	2	2	308	97
	4	3	1	126	97
	4	3	2	890	97
	62	1	1	n.s.	n.s.
	62	1	2	n.s.	n.s.
	62	2	1	n.s.	n.s.
	62	2	2	n.s.	n.s.
	62	3	1	n.s.	n.s.
	62	3	2	n.s.	n.s.
	Parental			13747	98
**F-Strain**	0	1	1	137	97
	0	1	2	91	97
	0	2	1	75	97
	0	2	2	69	97
	0	3	1	74	96
	0	3	2	108	97
	4	1	1	194	97
	4	1	2	206	97
	4	2	1	217	97
	4	2	2	377	97
	4	3	1	53	97
	4	3	2	305	97
	62	1	1	32	96
	62	1	2	46	97
	62	2	1	47	97
	62	2	2	96	97
	62	3	1	41	94
	62	3	2	409	97
	Parental			146263	98
**FR_sensitive**	0	1	1	111	97
	0	1	2	115	97
	0	2	1	120	97
	0	2	2	305	97
	0	3	1	95	97
	0	3	2	111	97
	4	1	1	77	97
	4	1	2	188	97
	4	2	1	26	96
	4	2	2	374	97
	4	3	1	90	96
	4	3	2	469	97
	62	1	1	107	97
	62	1	2	59	76
	62	2	1	37	97
	62	2	2	743	97
	62	3	1	9002	97
	62	3	2	168	97
	Parental			125137	98
**FR_resistant**	0	1	1	111	97
	0	1	2	115	97
	0	2	1	120	97
	0	2	2	305	97
	0	3	1	95	97
	0	3	2	111	97
	4	1	1	77	97
	4	1	2	188	97
	4	2	1	26	96
	4	2	2	374	97
	4	3	1	90	96
	4	3	2	469	97
	62	1	1	107	97
	62	1	2	59	76

	62	2	1	37	97
	62	2	2	743	97
	62	3	1	9002	97
	62	3	2	168	97
	Parental			74752	98

Virus	Line	Biological replicate		Mean coverage	% genome recovery
**K17+ (serial passaging)**	1	1		75271	98
	1	2		31756	97
	1	3		75948	97
	2	1		59078	97
	2	2		33047	97
	2	3		15912	97
	3	1		18933	97
	3	2		84879	97
	3	3		92606	97
	4	1		92991	97
	4	2		80867	97
	4	3		24841	97
	5	1		93078	97
	5	2		44605	97
	5	3		40911	97
	6	1		41820	97
	6	2		86495	97
	6	3		79662	97
	7	1		75322	97
	7	2		64724	97
	7	3		63316	97
	8	1		62870	97
	8	2		39562	97
	8	3		45734	97
	9	1		71590	97
	9	2		38103	97
	9	3		62347	97
	10	1		13031	97
	10	2		31896	97
	10	3		27851	97
	11	1		16438	97
	11	2		53726	97
	11	3		58356	97
	12	1		63851	97
	12	2		74823	97
	12	3		31841	97
	13	1		40139	97
	13	2		64837	97
	13	3		65095	97
	14	1		67229	97
	14	2		12096	97
	14	3		69155	97
	15	1		59058	97
	15	2		37581	97
	15	3		58850	97
