# Supplementary material for: Low frequency variants can predetermine antiviral drug resistance development in herpes simplex virus type 1
Source: PLoS Pathog. 2026 Jun 8;22(6):e1014296. doi: 10.1371/journal.ppat.1014296 (PMC13262928; doi:10.1371/journal.ppat.1014296)
Supplement: S2 Table — The numbering of each replicate corresponds to the biological replicate (first number) and technical replicate (second number). Resistance was determined based on previously published data (https://doi.org/10.5281/zenodo.14351987). (PDF) [file ppat.1014296.s002.pdf]

Gene	Mutation	ACV sensitivity	Virus	Parental Frequency (%)	Concentration	Replicate	Frequency (%)
UL23	T287M	Resistant	K17+ BAC	Not detected	4 µM	1.1	76,54
						2.1	10,80
			FR sensitive	Not detected	4 µM	1.1	35,38
						3.1	75,58
	T245M	Resistant	K17+ BAC	Not detected	4 µM	1.1	18,37
			FR sensitive	Not detected	4 µM	2.1	48,48
			FR resistant	7,43	4 µM	1.1	46,88
						1.2	39,33
						2.1	33,59
						2.2	41,86
						3.2	40,74
					62 µM	1.1	72,92
						1.2	71,67
						2.1	43,46
						2.2	63,03
						3.1	61,02
						3.2	52,45
	P355fsx	Resistant	K17+ BAC	0,16	4 µM	1.2	78,45
						2.1	24,26
						2.2	39,63
						3.1	80,37
	E146fsx	Not described	K17+ BAC	0,19	4 µM	1.2	9,31
						2.1	55,59
			F-Strain	1,67	4 µM	1.1	86,49
						1.2	89,16
						2.1	77,19
						2.2	62,25
						3.1	78,57
						3.2	70,86
					62 µM	1.1	82,14
						1.2	100,00
						2.1	84,44
						2.2	85,00
						3.1	87,88
						3.2	89,84
	G200D	Resistant	K17+ BAC	Not detected	4 µM	2.2	45,00
			FR sensitive	Not detected	62 µM	3.1	83,88
	E83K	Resistant	K17+ BAC	Not detected	4 µM	3.1	11,84
	P84S	Resistant	F-Strain	1,02	4 µM	1.1	63,39
						1.2	58,37
						2.1	72,53
						2.2	42,47
						3.1	58,62
						3.2	61,36
					62 µM	1.1	61,11
						1.2	20,59
						2.1	82,00
						2.2	69,05
						3.1	52,83
						3.2	48,02
	Q185fsx	Not described	F-Strain	0,13	4 µM	2.2	25,27
			FR sensitive	0,07	4 µM	2.1	37,04
						3.2	83,90
					62 µM	2.1	28,00
			FR resistant	1,23	62 µM	2.2	18,79
						3.2	23,91
	R216H	Resistant	FR sensitive	Not detected	4 µM	1.1	58,89
	R220C	Resistant	FR sensitive	Not detected	4 µM	1.2	95,45
	G356fsx	Not described	FR sensitive	0,05	4 µM	2.2	88,26
	R51W	Not described	FR sensitive	Not detected	62 µM	1.1	83,61
	A133fsx	Not described	FR sensitive	Not detected	62 µM	2.1	44,00
	Q342*	Not described	FR sensitive	Not detected	62 µM	2.2	96,46
	R256W	Resistant	FR sensitive	3,93	62 µM	2.2	94,34
	R163H	Resistant	FR resistant	2,69	4 µM	2.1	22,22
					62 µM	2.1	35,40
UL30	A1099T	Sensitive	FR sensitive	Not detected	4 µM	2.1	52,38
	V715A	Not described	FR resistant	35,94	0 µM	2.2	14,51
					4 µM	1.1	15,09
					62 µM	1.1	60,71
						2.2	66,32
	T821M	Resistant	FR resistant	1,92	62 µM	3.1	21,43
