# Supplementary figures and images for: Low frequency variants can predetermine antiviral drug resistance development in herpes simplex virus type 1
Source: PLoS Pathog. 2026 Jun 8;22(6):e1014296. doi: 10.1371/journal.ppat.1014296 (PMC13262928; doi:10.1371/journal.ppat.1014296)

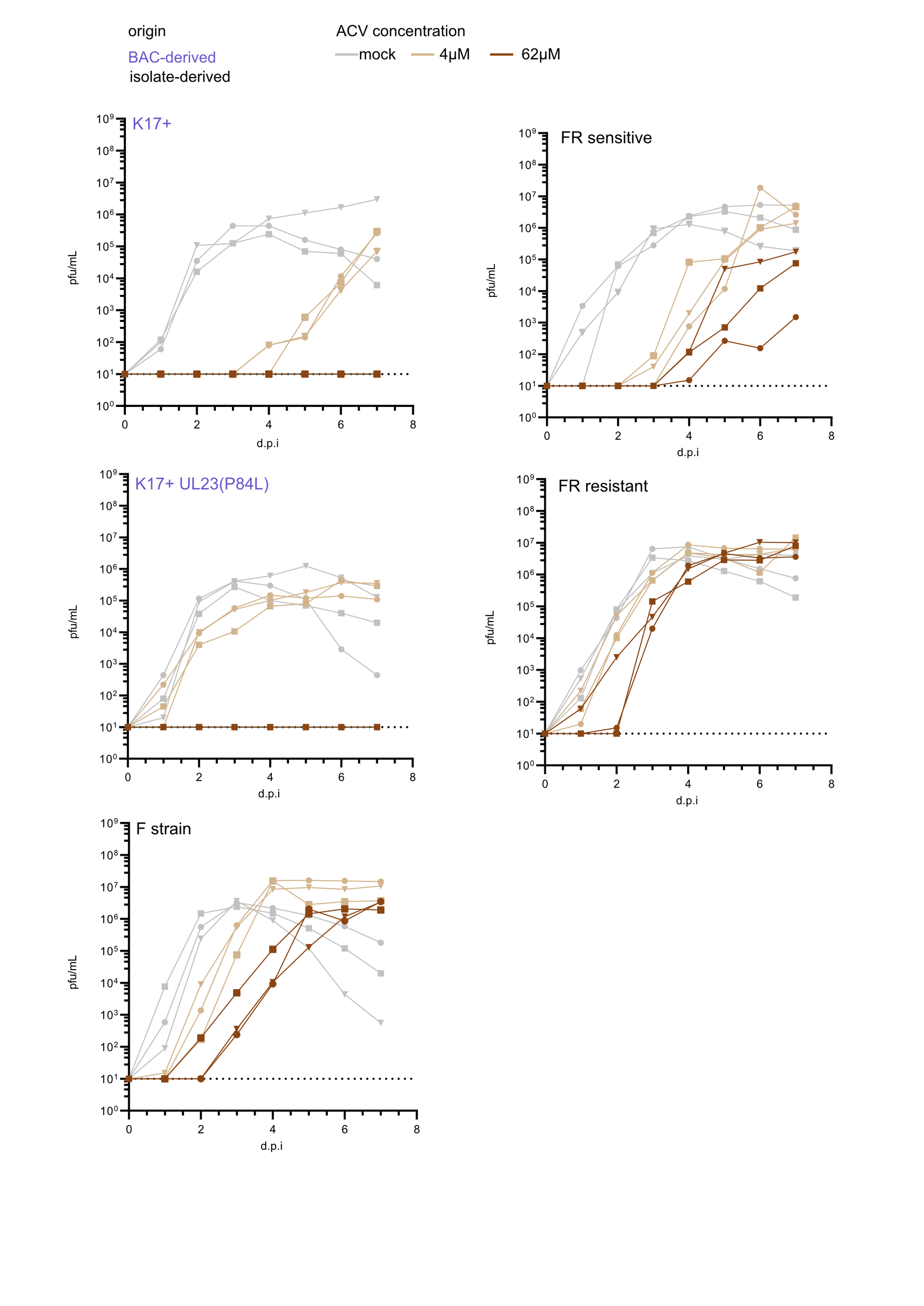

Supplement: S1 Fig — Vero cells were infected at a moi of 0.001 with the different viruses in the absence (mock) or presence of 4 µM or 62 µM ACV. Viral growth of the individual viruses was measured in biological triplicates by plaque assay. Each connected line represents one biological replicate. This Fig uses the same data as Fig 1. (TIF) [file ppat.1014296.s003.tif]

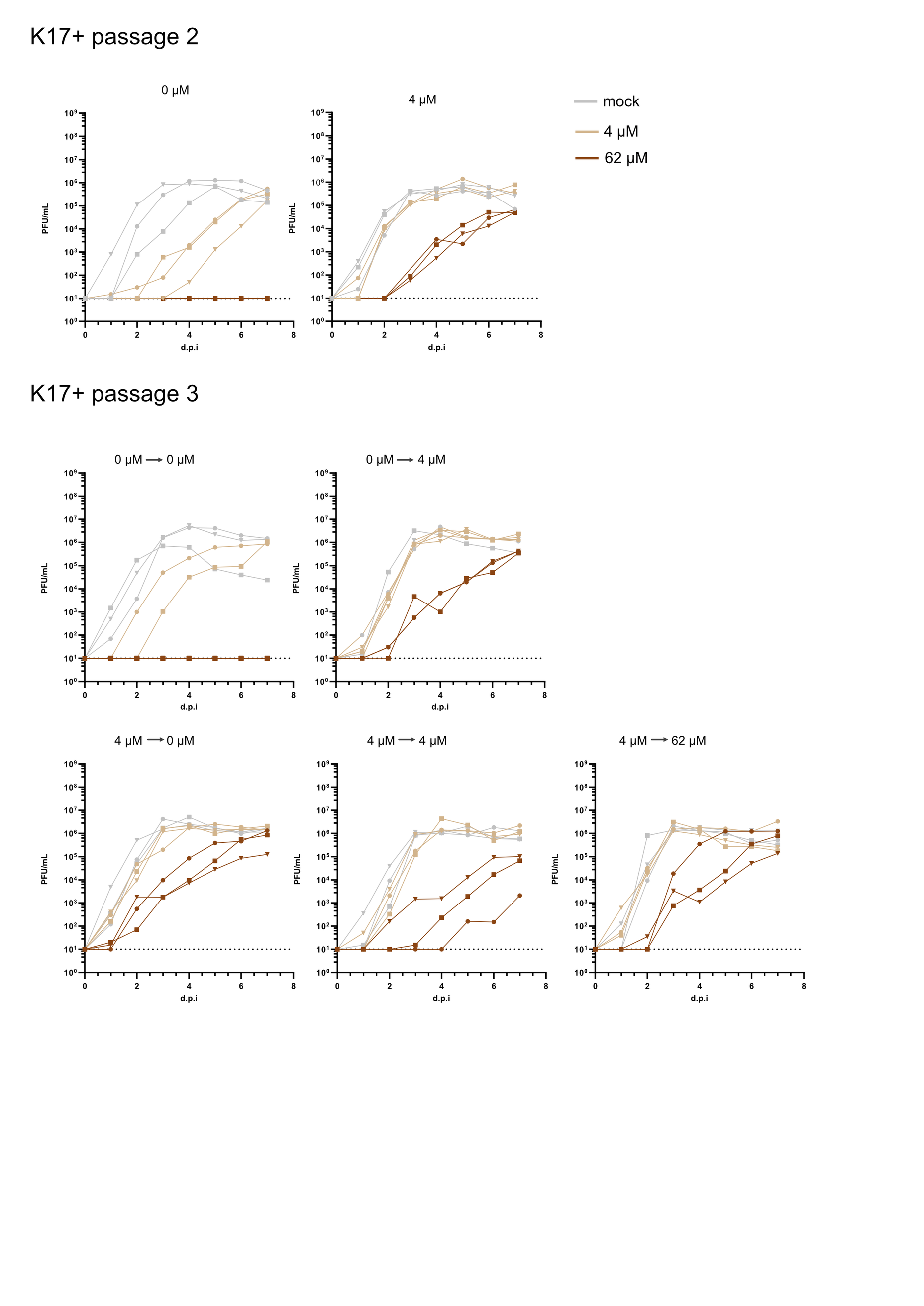

Supplement: S2 Fig — Vero cells were infected at a moi of 0.001 with the K17 + passaged viruses under different ACV concentrations in the absence (mock) or presence of 4 µM or 62 µM ACV. Viral growth of the individual viruses was measured in biological triplicates by plaque assay. Each connected line represents one biological replicate. The header above each Fig shows the prior passaging history. This Fig uses the same data as Fig 2. (TIF) [file ppat.1014296.s004.tif]

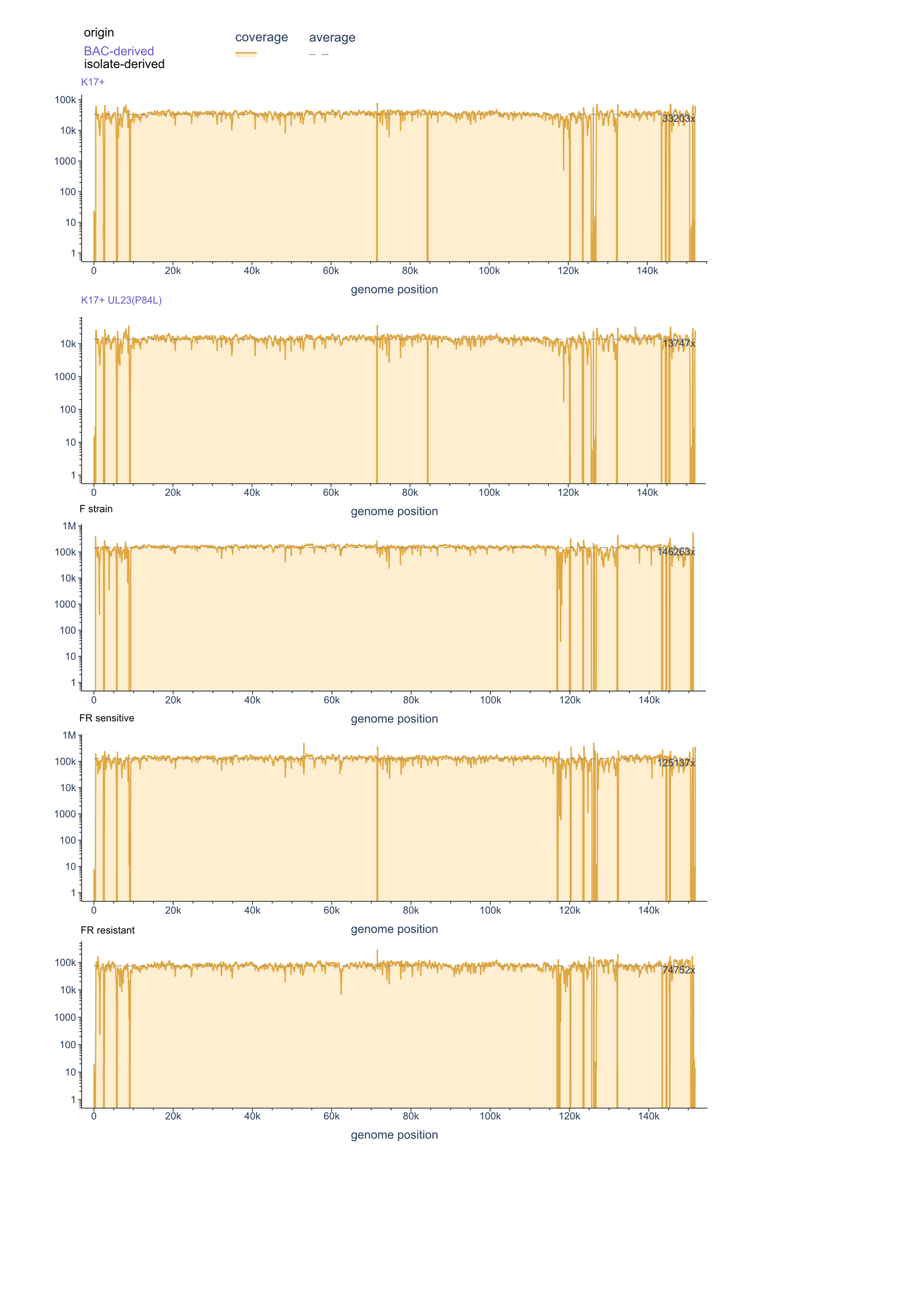

Supplement: S3 Fig — Parental viruses were de novo assembled and reads remapped to the newly assembled genome. Coverage plots were created with BAMdash. Below the coverage, the coding sequences of the individual reference genes are plotted. The dotted lines depict the mean coverage. (TIF) [file ppat.1014296.s005.tif]

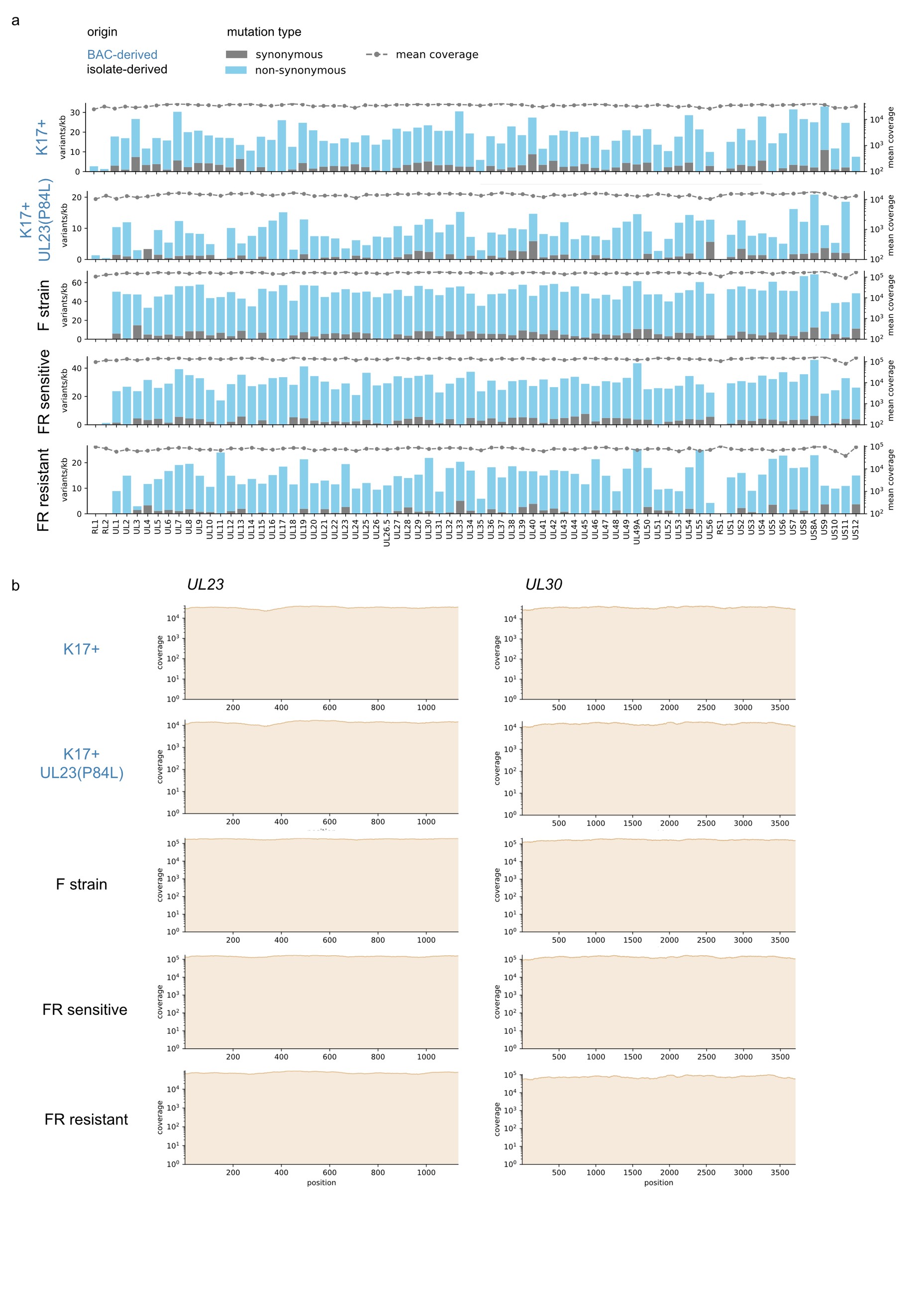

Supplement: S4 Fig — (a) Stacked bar plots showing for each parental virus the number of synonymous and non-synonymous variants/kb (left y axis) and the respective coverages per gene (right y axis). (b) Per-base coverage plots for both UL23 and UL30 of each parental virus. (TIF) [file ppat.1014296.s006.tif]

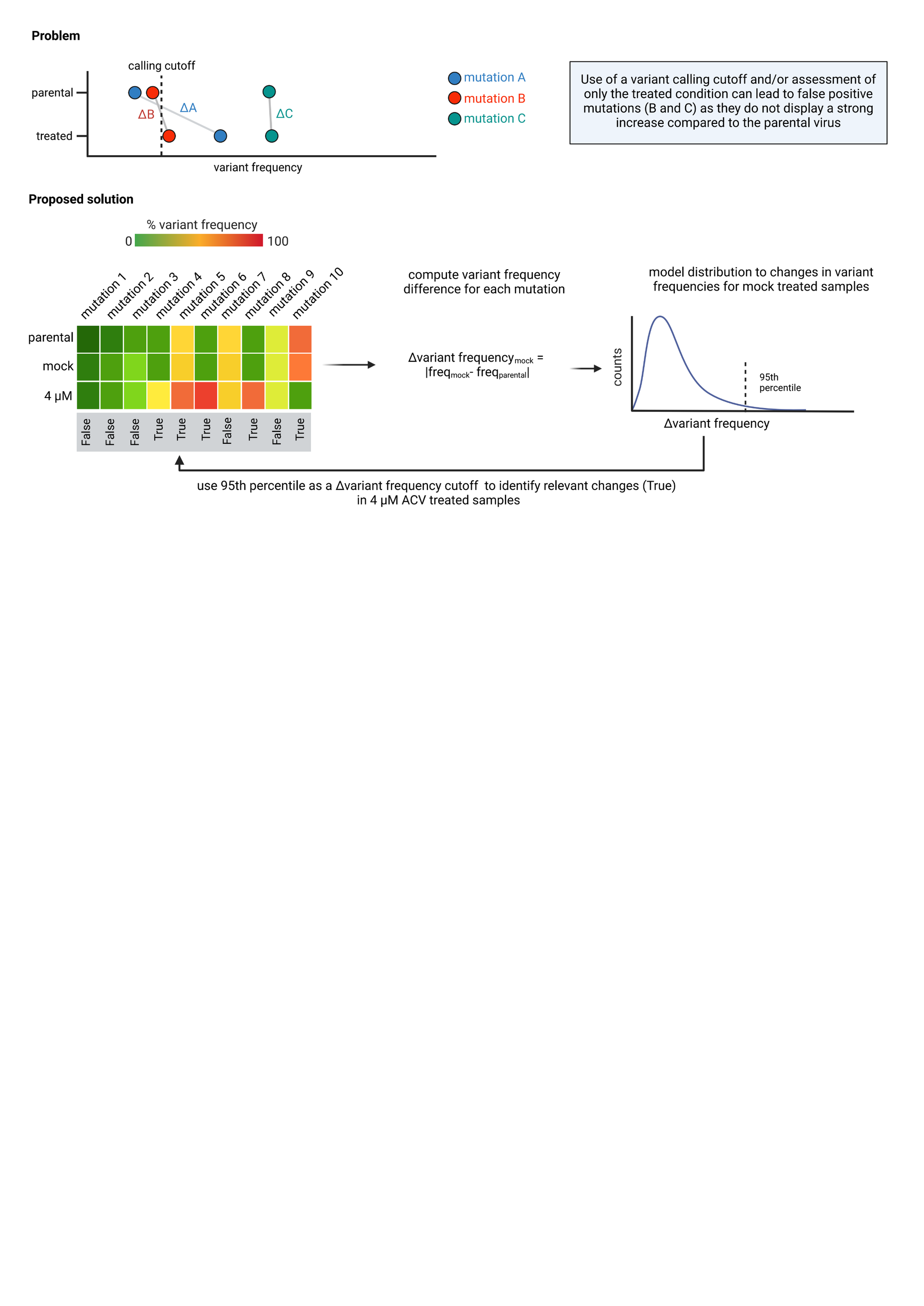

Supplement: S5 Fig — Rationale on how to identify mutations that are likely attributed to selection during treatment. Using variant cutoffs such as 1, 5 or 10% to find such mutations can lead to edge cases where mutations in the parental virus are slightly below the variant calling cutoff and slightly above the cutoff in the treated samples. Therefore, such mutations only have a marginal increase compared to the original mutation (mutation B). Moreover, a comparison between parental and treated viruses is important independent of a variant frequency cutoff to assess if mutations actual change in their frequency (mutation A) or not (mutation C). The here proposed solution is to call variants in the absence of a variant cutoff and instead focus on relevant changes during treatment. To define relevance, Δvariant frequencies are calculated and for the mock treated condition a distribution is modelled to define the 95th percentile above which mutations have a higher change in frequency than expected. We propose that in a setting with ACV treatment using this Δvariant frequency cutoff will lead to the identification of mutations that change significantly in their frequency. Created in BioRender. Fuchs, J. (2026) https://BioRender.com/6ya2i42. (TIF) [file ppat.1014296.s007.tif]

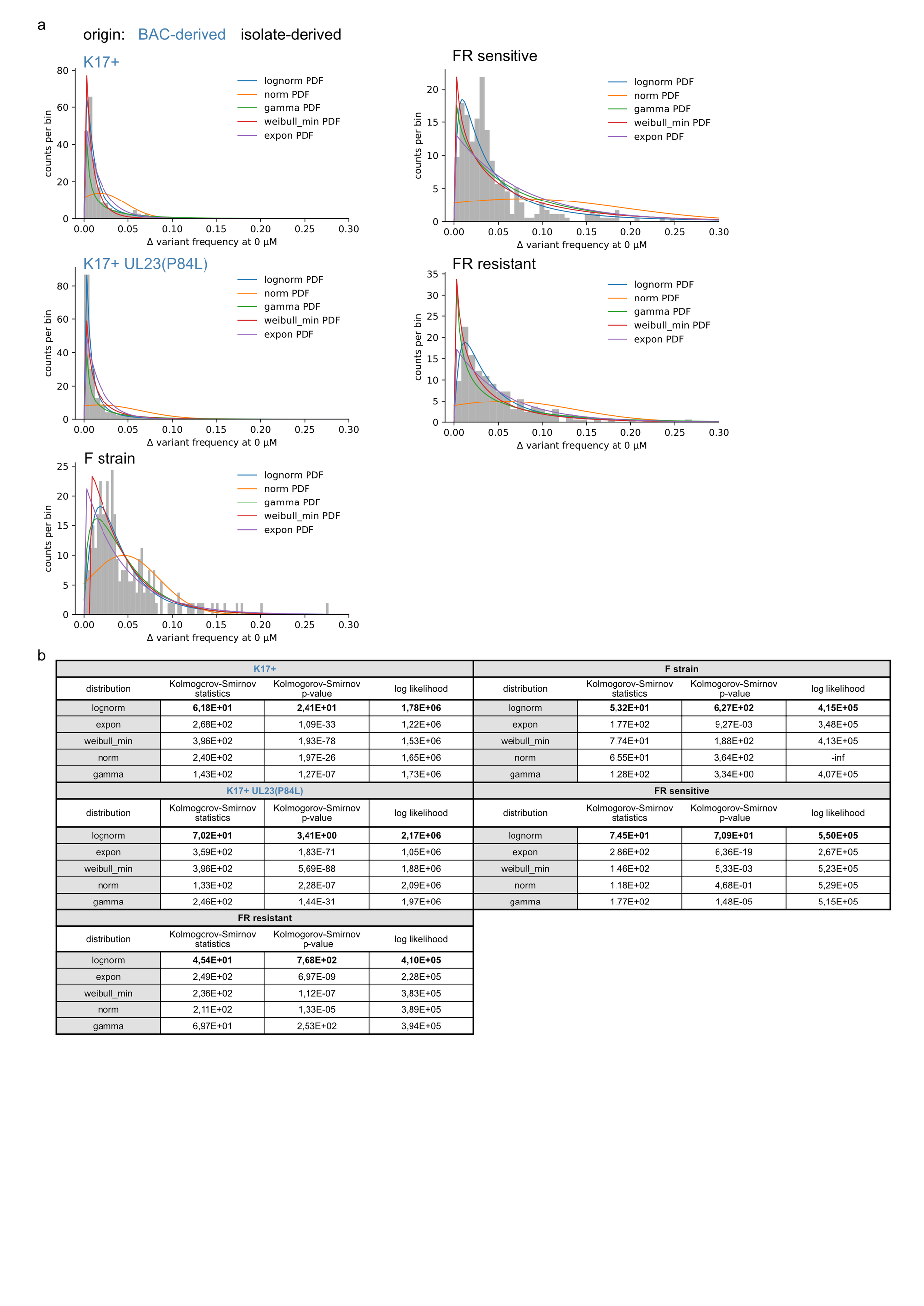

Supplement: S6 Fig — To assess the mutational background noise expected in the absence of ACV selection pressure, the cumulative Δ variant frequencies were evaluated for each mock-treated strain separately as the observed mutations and their respective frequencies were dependent on the parental virus stocks. (a) Therefore, different distributions were fitted to the Δ variant frequencies of the PBS treated control: log-normal distribution (lognorm PDF), normal distribution (norm PDF), gamma distribution (gamma PDF), Weibull distribution (weibull_min PDF) and exponential distribution (expon PDF). Δ variant frequencies are the absolute difference between the variant frequency of a mutation in the parental virus and the treated virus. Non-detected variants in the parental viruses were treated as having a variant frequency of 0. PDF – probability density function. (b) Distribution fitting was systematically evaluated by the Kolmogorov–Smirnov test and maximum likelihood estimation. Shown are the results for each test. Bold marked are the values that indicate the best fitting distribution for each test (Kolmogorov–Smirnov statistic: higher is better, Kolmogorov–Smirnov p-value: lower is better, log-likelihood: higher is better). For all viruses log-normal distributions fitted best to the Δ variant frequencies. (TIF) [file ppat.1014296.s008.tif]

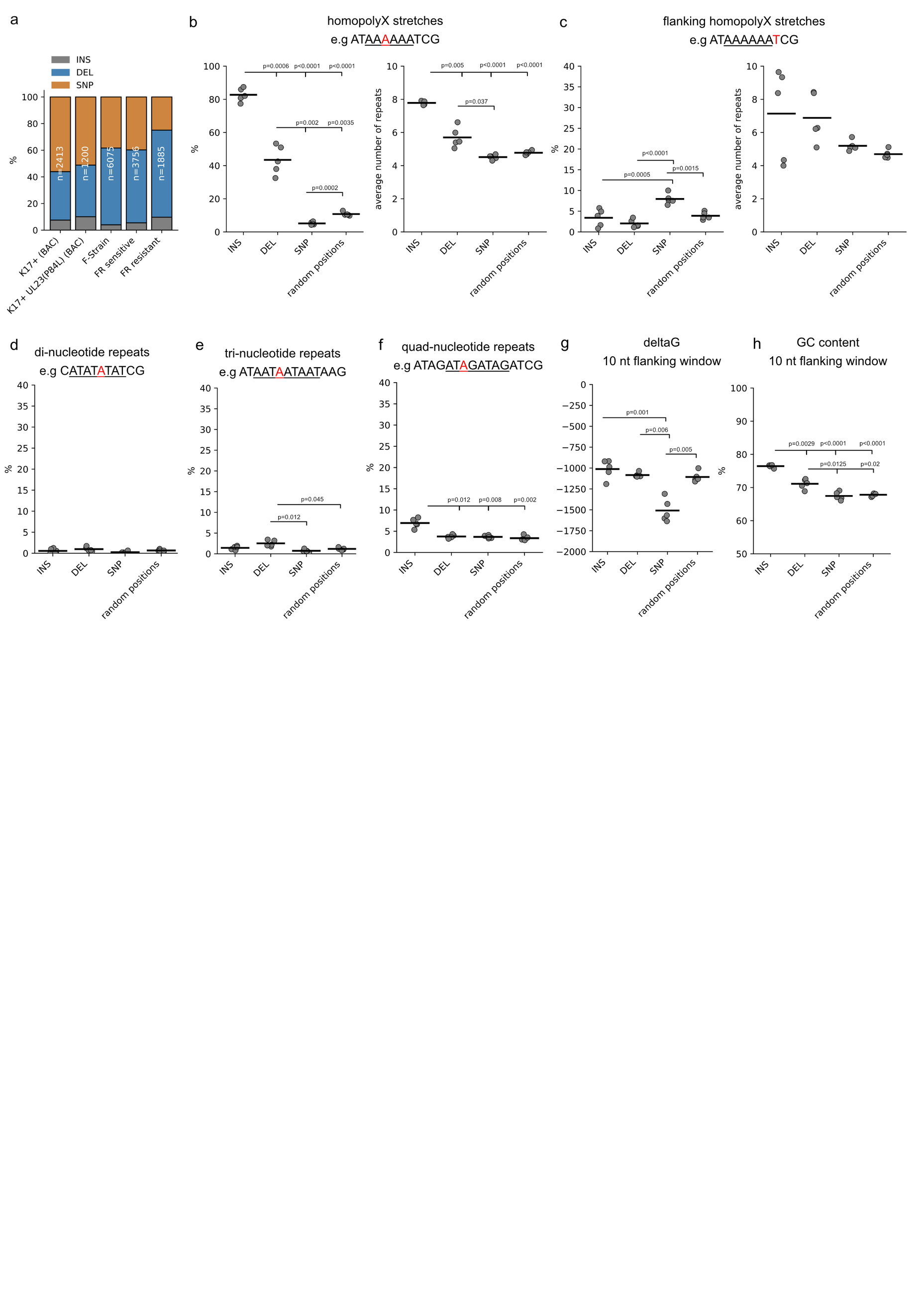

Supplement: S7 Fig — (a) Stacked bar plots depicting the percentage of insertions (INS), deletions (DEL) and single nucleotide variants (SNP) for each parental virus. (b-h) Using python3, sites of mutations were systematically evaluated for sequence patterns compared to 500 randomly selected positions in the viral reference sequence. Each dot represents the mean of all tested mutations per virus and substitution type. The horizontal lines indicate the mean. Examples for each pattern is given above the graph (underlined) with the site of mutation marked in red. (b) Percentage of mutations that were part of homopolymer stretches longer than 3 nucleotides (left) and the mean length of the detected stretch (right). (c) Percentage of mutations that had flanking homopolymer stretches longer than 3 nucleotides (left) and the mean length of the detected stretches (right). (d-f) Percentage of mutations that were part of di-nucleotide (d), tri-nucleotide (e) or quad-nucleotide repeats (f) of at least 4, 3 and 2 repeats, respectively. (g) GC content and (h) deltaG (left) of the flanking sequence region consisting of 10 nucleotides up- and downstream of the mutation’s regions were analyzed. (b-h) Equality of variances were tested with Levene’s test. Statistics were calculated with a one-way ANOVA together with Tukey multiple comparison test (c – left, f) for equal variances and Welch’s ANOVA together with Games-Howell multiple comparison test (remaining Figs). Multiple testing was only performed for significant ANOVA results (p ≤ 0.05). P-values from the multiple testing are indicated for p ≤ 0.05. (TIF) [file ppat.1014296.s009.tif]

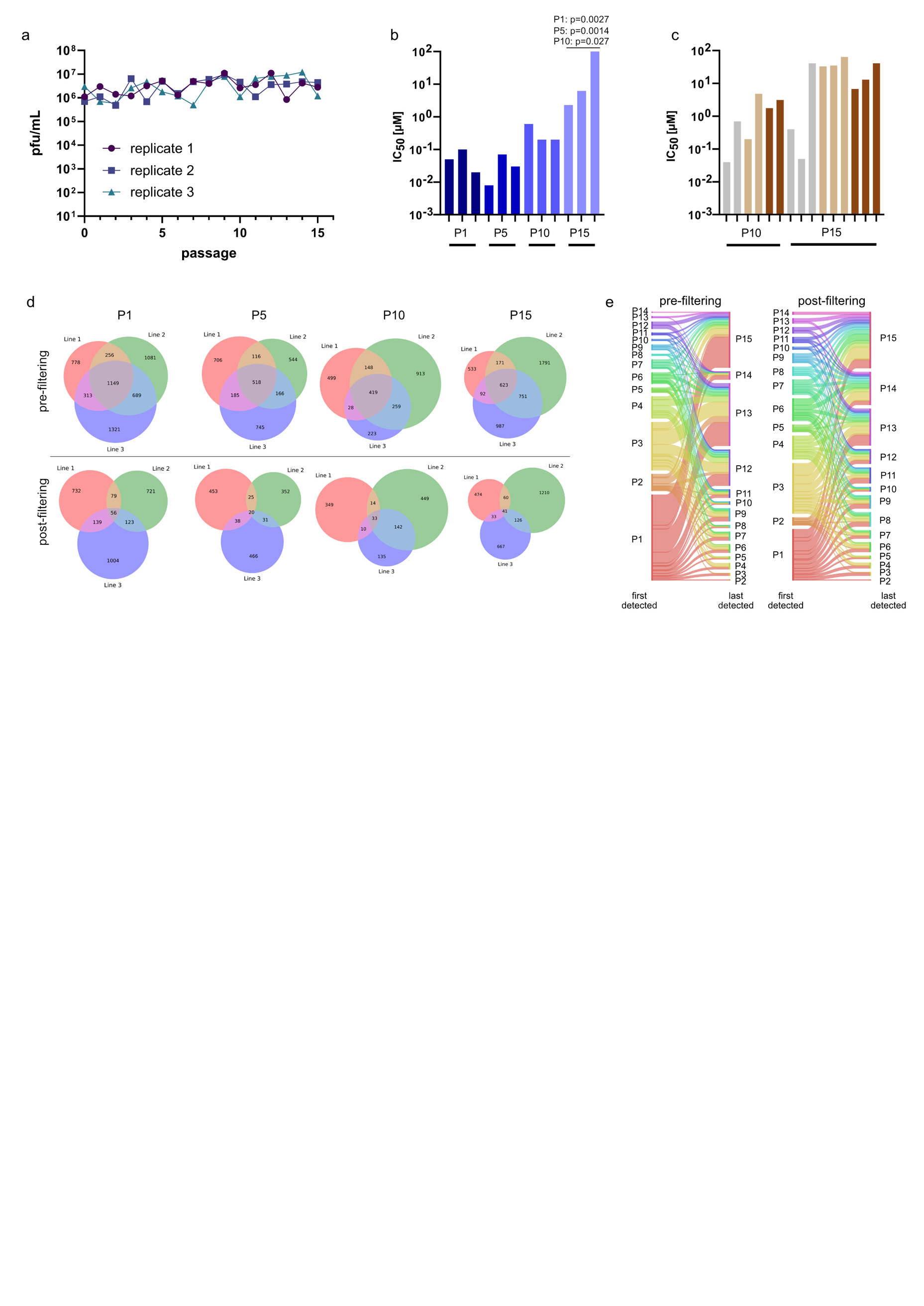

Supplement: S8 Fig — (a) Viral titers of each passage after 48 h post infection with 10 µl/well of a 6-well plate of the previous passage. Connected dots represent one consecutive passage. (b) IC50 values of passage 1, 5, 10 and 15 without selection pressure. Statistics were calculated with a one-way ANOVA together with Tukey multiple comparison test. P-values from the multiple testing are indicated for p ≤ 0.05. (c) IC50 values after successful culturing the individual replicates of passage 10 and 15 in the absence (mock) or presence of 4 µM or 62 µM ACV for 7 days. (d-e) Passages were individually sequenced and variants called compared to the K17 + reference genome. Next, variants were tracked over passages and individual adaptation lines (pre-filtering). Common mutations, defined as mutations present in all lines and in one line in at least 13 passages, were excluded if they did not increase in their frequency in any line (post filtering). (c) Venn-diagrams showing the number of mutations overlapping pre- and post-filtering between lines. (d) Sankey plot showing in which passage a mutation first and last appeared pre- and post-filtering. (TIF) [file ppat.1014296.s010.tif]
