# Supplementary material for: Low frequency variants can predetermine antiviral drug resistance development in herpes simplex virus type 1
Source: PLoS Pathog. 2026 Jun 8;22(6):e1014296. doi: 10.1371/journal.ppat.1014296 (PMC13262928; doi:10.1371/journal.ppat.1014296)
Supplement: Attachment — Submitted filename: Response to Reviewers.pdf [file ppat.1014296.s013.pdf]

Additional Editor Comments:

Three experts have reviewed your manuscript and have provided detailed comments. One recommended minor revision, and two recommended major revision. Please address the comments and include a point-by-point response with the revised manuscript. Reviewers 1 and 2 take issue with the use of the term quasispecies, and I tend to agree with them. Thank you for submitting your work to PLOS Pathogens.

Response: *We would like to thank all reviewers for their thorough and productive feedback which helped us enormously in improving the manuscript. We did now refrain from using the quasi-species term throughout the manuscript. However, we adapted our discussion to emphasise the quasi-species nature of our findings, given that pre-existing low-frequency mutations allowed rapid adaptations to changing evolutionary pressures.*

Please find attached our point-by-point response.

Journal Requirements:

1) We do not publish any copyright or trademark symbols that usually accompany proprietary names, eg ©, ®, or TM (e.g. next to drug or reagent names). Therefore please remove all instances of trademark/copyright symbols throughout the text, including:
- ® on page: 14 and 16.

Response: *Removed the trademark symbols.*

2) Please upload all main figures as separate Figure files in .tif or .eps format. For more information about how to convert and format your figure files please see our guidelines: https://journals.plos.org/plospathogens/s/figures

Response: *Uploaded all figures as *.eps*

3) Some material included in your submission may be copyrighted. According to PLOSu2019s copyright policy, authors who use figures or other material (e.g., graphics, clipart, maps) from another author or copyright holder must demonstrate or obtain permission to publish this material under the Creative Commons Attribution 4.0 International (CC BY 4.0) License used by PLOS journals. Please closely review the details of PLOSu2019s copyright requirements here: PLOS Licenses and Copyright. If you need to request permissions from a copyright holder, you may use PLOS's Copyright Content Permission form.
Please respond directly to this email and provide any known details concerning your material's license terms and permissions required for reuse, even if you have not yet obtained copyright permissions or are unsure of your material's copyright compatibility. Once you have responded and addressed all other outstanding technical requirements, you may resubmit your manuscript within Editorial Manager.
Potential Copyright Issues:

- Figure 1A. Please confirm whether you drew the images / clip-art within the figure panels by hand. If you did not draw the images, please provide (a) a link to the source of the images or icons and their

license / terms of use; or (b) written permission from the copyright holder to publish the images or icons under our CC BY 4.0 license. Alternatively, you may replace the images with open source alternatives. See these open source resources you may use to replace images / clip-art:
- https://commons.wikimedia.org
- https://openclipart.org/.

Response: *The figure was created with Biorender. We now uploaded the license as an additional file and state in the figure legend how the figure was created. Biorender figures are licensed under CC BY 4.0.*

4) Please amend your detailed Financial Disclosure statement. This is published with the article. It must therefore be completed in full sentences and contain the exact wording you wish to be published.
State what role the funders took in the study. If the funders had no role in your study, please state: "The funders had no role in study design, data collection and analysis, decision to publish, or preparation of the manuscript.".
If you did not receive any funding for this study, please simply state: The authors received no specific funding for this work.

Response: *We now provide a separate Financial Disclosure Statement stating that the funding partners had no role in study design, data collection and analysis, decision to publish, or preparation of the manuscript*

Reviewers' Comments:

Reviewer's Responses to Questions

Part I - Summary
Please use this section to discuss strengths/weaknesses of study, novelty/significance, general execution and scholarship.

Reviewer #1:

The manuscript by Jaki et al aims to investigate how phenotypic resistance to HSV-1 antivirals (primarily acyclovir) develop on a genomic level. Through a series of carefully designed experiments, they observe a low-level 'quasispecies' phenomenon within HSV-1 populations that provide a backbone for developing antiviral resistance. The manuscript is well written and the conclusions are generally supported by the data presented.

Reviewer #2:

This manuscript by Jaki et al uses different starting stocks of herpes simplex virus 1 strains 17 and F, as the basis for an exploration of viral adaptation to aciclovir (ACV) exposure. HSV-1 strains 17 and F are classic experimental models for the study of HSV-1 (though here the authors rebrand strain F as a clinical isolate rather than a laboratory model strain). These strains are used either from the starting point of a BAC-clone (for strain 17) or a plaque-purified lab stock (strain F). In each case, the authors include "positive controls" of previously-described ACV-resistant variants of these strains for

comparison. The viral stocks are then subjected to serial passaging (i.e., experimental evolution) in Vero cells either with or without ACV present. A variety of creative extensions of these passaging experiments are applied. The key findings of the paper are that a sizable number of low-frequency “mutations” (variants) are present in all of the HSV-1 stocks that they examine, and that these mutations can serve as the seeds for rapid viral adaptation to achieve ACV resistance. The paper includes a solid experimental design, good replication of results, and a clear progression through their findings. Issues in need of attention include the methods for quantifying these low-frequency variants (i.e., absent or unclear quality control on variant calling), and the choice of language and interpretation (e.g., use of quasi-species, clinical isolate terms). These and other more minor recommendations are detailed below.

Reviewer #3:

In this manuscript, the authors analyzed the impact of ACV (acyclovir) selective pressure on different strains of herpes simplex virus 1 (HSV-1) in vitro. Following growth kinetics and IC50 evaluation, the authors concluded that development of ACV resistance occurred within a single passage. By using ultra-deep, non-targeted full-genome Illumina sequencing of the parental and ACV-adapted HSV-1 strains, ACV resistance mutations rapidly arose in the viral genes UL23 and UL30 and the authors claimed that they were already present in the parental ACV-naïve strains at extremely low variant frequencies. They hypothesized that low-variant frequencies represent an error-borne quasi-species swarm that develops during continued viral replication. They used a primary rescued recombinant K17+ strain and by continued passaging, an increased in the viral quasi-species pool was detected at low variant frequencies and allowed resistance development after 10 consecutive passages but not before. They deduced that viral quasispecies are a crucial prerequisite in the evolution of herpesviruses like HSV-1, enabling adaptation to selective pressures and that viral quasi-species assessment could allow prediction of a drug resistance potential. The authors have done a huge amount of work but some of the conclusions are well known (i.e., viral quasispecies are a crucial prerequisite in the evolution of herpesviruses) and others are quite controversial (i.e. rapid emergence of resistance after a single passage and the presence of ACV resistance mutations in the parental ACV-naïve strains at extremely low variant frequencies). Before this paper can be considered for publication, several points need to be clarified.

Response: *We would like to thank all reviewers for their time and effort reviewing the manuscript. We have addressed all of the comments below, and we believe that their constructive feedback has significantly improved the manuscript. As some of the points raised by the reviewers overlapped, we have numbered the points individually and referenced other reviewers where applicable, in order to avoid duplications in our responses.*

All line references are given for the manuscript file that has the tracked changes marked in red.

Part II – Major Issues: Key Experiments Required for Acceptance
Please use this section to detail the key new experiments or modifications of existing experiments that should be absolutely required to validate study conclusions.

Generally, there should be no more than 3 such required experiments or major modifications for a "Major Revision" recommendation. If more than 3 experiments are necessary to validate the study conclusions, then you are encouraged to recommend "Reject".

Reviewer #1:

(No Response)

<u>**Reviewer #2:**</u>

- <u>**Point 1:**</u> Use of the term "quasi-species" (in the title, abstract, summary, and main text) is not correct by definition, nor is the parallel helpful to the reader in the way it appears to be being used here. In virus evolution parlance, the term quasi-species is generally applied to RNA virus populations, where selection is postulated to act on a swarm of non-identical genomes that all carry mutations that could lead to lower fitness if they existed alone. In a viral quasi-species, the sum total of co-existing variants performs better than any single member, and could thus be maintained or constantly regenerated under evolutionary selection. Neither of these principles appear to apply to the observed data here, and nor are the relevant evolutionary aspects of quasi-species discussed in the manuscript or central to the results. Instead, the authors observed a very low number of HSV genomes that carry mutations, which co-exist with a very large percentage of non-mutant or intact genomes that dominate the viral population (e.g., most variants exist at frequencies <0.001). While the presence of the low-frequency variants is important, this viral population is not functioning as a quasi-species. The authors elsewhere use the terms "minority variants" (line 321), "low-variant frequencies" (line 339), and "low-frequency mutations" – any or all of which would work better throughout the manuscript (including title, abstract, summary, etc.).

  <u>***Response:***</u> *Thank you for raising these concerns as it is indeed controversial. We used the term quasi-species based on several observations. First, the observed low-frequency mutations can be relevant for stringent evolutionary bottlenecks, such as antiviral treatment, as they can be selected for over several orders of magnitude. Second, our seven-day growth kinetics experiments revealed distinct differences between different HSV-1 strains which are likely due to different levels of prior diversification (likely due to differences in passaging history – please also kindly refer to later points for clarification of passaging history and plaque purifications). Third, we found a maintenance and considerable increase of several mutations during the experimental evolution of the K17 strain in the absence of ACV treatment. This could be explained due to fitness benefits of a diversified population in cell culture. Altogether, we believe that the quasi-species concept, or at least some of its principles, can also be applied to HSV-1.*

  *Nevertheless, we agree that the reviewer's concerns are valid, as the relevance of the observed resistant low-variant frequency mutations to viral fitness is still unclear, and we do not provide sufficient proof that the quasi-species concept applies here. Further experimental verification of our claim is required, which is beyond the scope of this paper. Therefore, we refrained from using the term quasi-species throughout the manuscript **(line 1, 4, 37, 38, 40, 51, 53, 83, 219, 252, 260, 308, 310, 323, 324, 369, 387, 390)** but propose a quasi-species like 'character' of our observations in the discussion **(line 330-331)**.*

- <u>**Point 2:**</u> Quantification of low-frequency mutations – A key finding of the manuscript is its documentation of low-frequency mutations in the starting viral stocks, and its demonstration of how ACV-resistance mutations can be selected from this swarm or cloud of variation. The high allele frequencies achieved by the ACV-resistance mutations demonstrate that the overall

findings are valid. However the quantitation of low-frequency variants lacks sufficient rigor. The methods mention use of the fastp package for quality control of Illumina sequence reads, but no parameters are stated. After read mapping, it is standard practice to apply criteria for the detection of low-frequency variants (e.g., PMCID: PMC10120596). These would typically include a minimum variant frequency (e.g. 1-2%) minimum coverage depth (e.g., 50-100X), bidirectional read support, and a minimum number of reads supporting the variant allele (e.g., 9 in PMC10120596). None of these appear to be applied in this study. The top row of Figure 5C illustrates the outcome of applying no filters, with most of the >5,000 variants in each panel having a frequency below 0.1 – a level that likely includes noise and are rarely included in peer studies. Without rigorous quality control or the use of unique molecular identifiers (UMIs) during library preparation, it is not clear that these extremely rare variants should be included. The focus on “change in variant frequency” in Figures 3-4 obscures the rarity of these variants. Technically, a variant that has a measured frequency of 0.1 and rises to a level of 0.5 has changed by 5% -- but may be entirely within the level of noise. Without rigorous quality control or UMIs, the statistical approaches used to pick an acceptable frequency cutoff for each experiment (Supp. Fig 1) fall short of the standards recommended for variant detection in herpesviruses (e.g., PMC10120596).

Response: *We thank the reviewer for highlighting the missing details were missing regarding how variant filtering was performed in our study. We added further documentation of the specific parameter choices of the used software packages* ***(line 480-484)****, which are similar to other recent peer-reviewed studies with HSV-1 (e.g. https://doi.org/10.1093/molbev/msae119).*
The introduced metric Δvariant frequency aims to filter low-frequency variants as well as mutations that do not significantly change between the parental and treated virus populations if they are present in higher frequencies (the rationale for this is now graphically depicted in ***Supplementary Figure 5)****. It is computed for each mutation as:*

$$\Delta\text{variant frequency}_{\text{treated}} = \left|\text{variant frequency}_{\text{treated}} - \text{variant frequency}_{\text{parental}}\right|$$

The Δvariant frequency cutoff describes a rise in frequency that is unlikely under mock conditions. Therefore, this will reduce both the number of low-frequency mutations and the number of high-frequency mutations that remain unchanged. By default, this metric introduces even stronger variant frequency cutoffs than proposed by the reviewer. Figures 3-4 does not obscure rare variants, as rare variants are never included. They all have to exhibit a variant frequency between 5-25% (depending on the strain) if they were not initially detected in the parental strain. We extensively revised the results part to clarify this metric as this was not well described in the previous version of the manuscript ***(line 152-157, 170-188, 193-198).***

We would also like to state that our primary aim is to quantify low-frequency variants in order to assess whether mutations responsible for ACV resistance are pre-existing. The referenced publication PMC10120596 has a different objective: to quantify all high-confidence variants arising in HCMV, likely excluding not only sequencing errors but also true low frequency variants.
To address this issue, we applied the variant filtering scheme suggested in the aforementioned publication (PMC10120596) to our data using the Snakemake workflow. We then compared the detected variants.

Using this filtering scheme did not allow the majority of low-frequency variants (between 60-100%) to be detected, even though they increased in frequency by at least 5-fold during passaging (Figure 5). As some of these variants increased by several orders of magnitude and could become fixed during passaging, it is highly unlikely that they represent technical errors. ***Please also refer to Point 41****.*

- **Point 3:** Methods for sequence feature analysis – The methods for the analysis of sequence "regions that might cause problems" (line 224), and the related Supp. Fig. 6, are completely missing. While the idea of this sequence feature analysis is good, the chosen thresholds are generous and not explained. E.g., "homopolyX" (mono-nucleotide runs) are counted from as little as three of the same consecutive bases. Similar analyses often start at a level of 4. Other short repeats (e.g., di-, tri-nucleotides) are counted from a simple dyad on up. These generous settings inflate the number of instances detected, and should be justified if used. The right-hand side of each analysis panel displays the breakdown of these findings, and highlights the large number in each category that are at the "bare minimum" level of meeting the detection criteria. The left-hand panel uses the aggregate numbers to establish significance, and there is a concern that the large number of "bare minimum" loci may be inflating significance. This analysis would be more impactful if run with a more rigorous requirement for what constitutes each sequence feature.

  Response: *Thank you for addressing these issues. We would like to emphasise that we provide the full code which we used for our data analyses including the feature sequence analysis (see Data availability statement and kindly refer to the check_seq_patterns() definition in helper_functions.py in the provided github repository). To clarify, we now state in the* ***figure legend*** *that this was analysed with python3.*

  We want to further thank the reviewer for the suggestion of using more stringent settings. For the repeats, we adjusted our analysis of dinucleotide repeats (3 minimum repeats, e.g. ATATATAT), trinucleotide repeats (minimum 2 repeats, e.g. ATCATCATC) and quad-repeats we left unchanged (ATCGATCG). One primary issue we now encountered was that the raw number of detected repeats per virus and mutation type were in some cases extremely low or even 0. This was anticipated as our previous analysis showed that the mean repeat length was in all cases just slightly above 1. The consequence of the more stringent analysis is that the analysis of the repeat length in the right figures is misleading as some datapoint are supported by a low number of actual mutations which creates an inherent data bias. Therefore, we excluded the right figure from the repeat length analysis and only presented the frequencies. We did not originally interpret any significant differences in the repeating regions because the mean repeat length was similar in all cases. The updated analysis now makes it clearer that repeats are not as relevant in our cases, as they account for only a small percentage of the detected mutations. This was an excellent suggestion which simplifies data interpretation.

  Similarly, we changed homopolymer stretches to a minimum length of 4 which also improved interpretability as it resulted in slightly more pronounced differences. Please refer to ***Supplementary Figure 7*** *and its Figure legend and our adjusted text* ***(line 244, 248).***

- **Point 4:** Figure 1 / experimental design – it would be useful to include an estimate of the starting viral population in addition to the MOI (i.e., calculated from volume of actual dish(es) used for

infection). This is relevant because at an MOI of 0.001, the total PFU input dictates the potential rarity of introducing very rare mutants from the starting population. By stating the PFU, it helps the reader understand the size of the starting population, and provides a more straightforward comparison with other experimental-evolution studies.

Response: *Thank you for the suggestion, we now include the number of infectious particles in the descriptions of Figure 1 and Figure 2* ***(lines 674 & 691).***

- **Point 5:** HSV-1 strain F was isolated from a patient lesion originally, but it has since been propagated in many laboratories for many passages, and it is widely considered to be a reference or laboratory strain (e.g., PMCID: PMC516408, PMC7764767). For the HSV field, the term "clinical isolate" usually refers to recent patient isolates of a known and/or low passage number. This is not the case for strain F, and the text should be adjusted accordingly (e.g., lines 108, 111, & throughout).

  Response: *The reviewer is right. As all used strains (even our in-house isolated strains) have been passaged many times, we refrained from using the term "clinical isolate" throughout the manuscript.*
  In the figures we now call them isolate-derived analogous to BAC-derived. We adjusted the text in ***lines 86, 106, 110, 113, 147, & 673*** *accordingly.*

Reviewer #3:

- **Point 6:** A major issue regarding this study is the choice of cells if one wants to extrapolate the results to the clinic. Vero cells are a continuous (immortalized) lineage of African green monkey kidney epithelial cells. They are aneuploid and are interferon-deficient, which may impact the time to select drug-resistant viruses. Thus, selection of drug-resistant viruses should be more relevant in primary human cells (either fibroblasts or epithelial cells) where HSV-1 easily grows. Therefore, the conclusions the authors drawn from their study should be much more cautious.

  Thank you for raising this issue. We agree that conclusions from Vero cells should indeed be more cautiously phrased. At the moment a large majority of diagnostic laboratories rely on Sanger Sequencing and might miss mixed virus populations. To the best of our knowledge, the clinical relevance of mixed and partly resistant populations is not well studied for HSV-1. Whether or not resistance conferring low-frequency-mutations are relevant in clinical/diagnostic practice has to be assessed.

  Response: *Our intention was not to oversell our results, rather we wanted to provide an insight into why we believe that NGS-based analyses of HSV-1 resistance status could improve patient care. We apologize for the extrapolation and tuned down/rephrased the clinical implications of our study throughout the text* ***(lines 40, 53-54, 81, 92-95, 370, 379-381, & 389-392).***

- **Point 7:** Line 151, "We were able to reconstruct highly covered viral genomes with mean coverage of up 152 to 100.000-fold (Suppl. Fig. 3)." There are quite some differences in coverage along the HSV-1 genome, with some regions showing very low coverage. What is the coverage along the genes of interest UL23 and UL30? Suppl. Fig 4 shows the mean coverage per gene but

not the variations in coverage along the UL23 and UL30 genes. Different individual positions of interest could exhibit deviating coverages.

<u>Response:</u> *Thank you for addressing this point. The coverage dips are not in coding UL or US regions but in the TRS, IRS and IRL regions that are inherently difficult to map with short read techniques due to long repeating regions. We are not aware of any technique that would allow precise mapping for short read technologies in such regions. The UL/US coverage, which contains most of the coding genes, is quite homogenous. We have now included per-base coverage plots for UL23 and UL30 for all parental viruses* ***(Supplementary Figure 4 b)*** *and mention it in the text in* ***lines 163-164****.*

- **<u>Point 8:</u>** What is the False Discovery Rate (FDR) cut-off? Strict threshold should be used to ensure high confidence in called variants (1% to 5%).

  <u>Response:</u> *This strongly relates to* ***Point 2 of Reviewer 2****; we kindly refer to our response for a more detailed technical explanation and line references. In brief, we did not use strict frequency cutoffs during variant calling as our idea was to assess if relevant (selection-attributed) mutations can be found in the parental virus which we likely miss with a defined variant cutoff. Moreover, a stringent variant cutoff at a coverage of 100.000 at 1 % would have excluded variants that are still supported by 1.000 reads. However, we are aware that with our relaxed settings, we likely also call some variants that might not truly exist due to e.g. sequencing or mapping errors. It is not easy to calculate an FDR rate and instead we rely for this solely on the variant caller lofreq that was particularly made for such cases.*
  A central concept of this study is that we focused on mutations that change in their frequency which fundamentally opposes the use of a fixed variant cutoff. This concept is applied in our delta frequency approach (kindly refer to our new ***Supplementary Figure 5*** *that graphically explains this rationale). Here, we do perform frequency filtering: for example, a delta variant frequency cutoff of 0.05 means that a mutation has to be present in at least a frequency of 5 % if it is not detected in the parental virus. If it is detected in the parental virus in e.g. 10 %, its frequency after adaptation has to be at least 15 %. Similarly, in our experimental evolution setup (Figure 5), our focus is ultimately on mutations that increase by at least 5-fold over multiple passages. Therefore, we initially do not use a variant cutoff but in later filtering steps we are far more stringent than a standard 1-5% cutoff. Notably, this is only possible due to our unique experimental setups and is likely not applicable for most use-cases.*

- **<u>Point 9:</u>** The results should be presented in a more clear way to facilitate the reading of the manuscript. For instance, which mutations did emerge following ACV pressure (Fig. 1)? Where these mutations detected in the parental virus? Figure 4 shows the mutations conferring resistance in UL23 and UL30 already present in the viral stocks but it's not specified in which parental virus there were present and under which condition they were selected. Which ACV-resistance mutations were acquired by the each parental virus?

  <u>Response:</u> *Thank you for pointing this out. Visualizing this for different viruses and a multitude of mutations represented a major challenge and we do not have a perfect solution to visualize all parameters in one figure. Therefore, we now include a table with all UL23/30 mutations over our cutoff, in which frequency they were present in the parental virus and to which frequency they*

*expanded in the respective replicates of each experimental condition (see Supplementary Table 2, now **referenced in line 257**). For the parental viruses (Figure 4 a and b) we would like to refrain from providing a table of all detected mutation (in total 300 individual mutations), instead we provide a **Supplementary data excel file**, showing which mutations were detected in which viruses **(referenced in line 224**). All raw data for all detected variants is also given in the provided github repository.*

- **Point 10:** Fig. 1b. There are quite some differences in sensitivity to ACV among the HSV-1 viruses that are sensitive to ACV. The K17+ (BAC-derived) has a substantial lower IC50 value than the F-strain and FR_sensitve isolate. This will certainly impact the fold-resistance.

  Response: *The reviewer is correct, there are strain specific differences in IC_{50} values. To the best of our knowledge this is a common observation. Based on our data, a cautious hypothesis would be that this is due to the differences in the prior diversification of the viral stocks, with a more homogenous viral population being more sensitive if it does not have an already fixed resistance mutation. This is for example supported by the continued passaging experiment where the K17 strain became more resistant even though it was never exposed to ACV (Suppl. Figure 8). For a mixed population with resistant subpopulations, we would hypothesize that a plaque reduction assay might cause selection, e.g. a virus population with 10 % resistant viruses might be selected during plaque reduction assays with the 10 % being the plaques that appear at higher ACV concentrations. So, the overall virus population might appear more resistant overall, but the effect actually just stems from the resistant subpopulation. However, this hypothesis would need additional investigations that are beyond the scope of the current manuscript.*

- **Point 11:** It is very hard to understand why ACV resistance mutations would develop and increase over consecutive passaging in the absence of ACV pressure. The authors should demonstrate that these data are reproducible that there are no errors related to detection of minor viral variants.

  Response: *Thank you for addressing this point. This relates to previous points **(Point 2, 8 and 10)**. We were actually surprised as well, but we would like to emphasise that the data has been reproduced in independent triplicates. One explanation for this finding could be that the viral TK is not required for viral replication in cell culture and that mutations such as frameshifts are more easily tolerated and/or there might be a fitness benefit for mutations increasing by at least 5-fold in our experimental setting. We discussed this in **lines 366-367.***

- **Point 12:** If ACV resistance would emerge so easily, it should occur much more frequently in the clinic than what is reported.

  Response: *Thank you for pointing this out. The resistance development in patients is indeed more complex than in cell culture as immune responses and tissue specific milieus, in tandem with antiviral treatment, are likely to play a role. However, in heavily immune-compromised patients, such as stem cell transplant recipients, antiviral resistances are frequently observed and can reach up to 46 % of cases (Tali Shafat et al Clinical Microbiology and Infection 2025, https://doi.org/10.1016/j.cmi.2025.01.033). So, while resistance development is overall low, it can be high in specific groups of patients **(please refer to line 67).***

- **Point 13:** Fig 5 is very difficult to follow. Is it correct that most of the mutations detected when passaging the viruses are not detected at #15? Which viral variants emerged as the dominant ones and are related to ACV resistance?

  Response: *As we detected a multitude of mutations in each passage, we wanted to know if these mutations are maintained or if each passage generates essentially a new genotype. What we observed was that a lot of mutations are detected in all lineages and passages but do not change in their frequency. We performed then exclusion of such positions (this included low and higher frequency mutations – kindly also refer to* ***Point 41****). What we deduced from the remaining mutations is that newly emerging mutations are maintained for a couple of passages but also disappear in regular intervals (Figure 5d). Therefore, the unique genotype of each passage is based on mutations from all previous passages. Newly appearing mutations from a current passage are then also again maintained for a couple of consecutive passages. This filtering step is descriptive and not necessarily needed* ***for Figure 5 e (now Figure 5 c) and 6 (now Figure 5 d)*** *as our focus here is on mutations that increase in their frequency. However, we show that passaging continuously changes the genotype in a consistent way (this is not trivially deduced from the large quantity of mutations we actually detected in each passage). We realized that it is was not well described in the manuscript. Therefore, we moved c and d to supplements and adjusted the text to clarify this rationale* ***(lines 277-295).***

  Your second question is answered by Figure 5c and d. We next focused on mutations that increase or decrease in their frequency as we hypothesized that these are the most relevant ones for the observed phenotype. This was only a small subset of all mutations (Figure 5c). If we then analysed these mutations, we indeed find mutations that are published resistance mutations or frameshifts in UL23 (Figure 5d). Therefore, we propose that these increasing mutations form the genetic basis of the observed phenotype. We moved figure 6 now to figure 5 to make it clearer that they belong together.

- **Point 14:** The authors should be careful with the statement 'viral quasi-species assessment could allow prediction of a drug resistance potential'. There is a general concern in the scientific community on the clinical significance of low-prevalence mutations (detected by NGS at a prevalence between 5 and 15%). It is crucial to analyze patients with low-prevalence mutations and how they respond to treatment over time to define the potential significance of minor variant populations. Besides, not all mutations detected at low frequency will emerge as the domini ant variant since they may have a high fitness cost, which will be influenced by the drug selective pressure. The authors should precautious in giving recommendations based only in in vitro studies.

  Response: *Thank you for addressing this. This concern relates to the prior* ***Point 6****. Please refer to the response to this earlier point for more details and line references. We have addressed this throughout the manuscript and tuned down our interpretation/clinical extrapolation.*

Part III – Minor Issues: Editorial and Data Presentation Modifications

Please use this section for editorial suggestions as well as relatively minor modifications of existing data that would enhance clarity.

Reviewer #1:

My only significant critiques of the work are as follows:

- **Point 15:** 1. I do not think the experiments definitively prove that the development of resistance requires the presence of pre-existing low frequency mutations (lines 95-97). This can mostly be addressed by simply softening the language used to state that their data supports this as one mechanism by which antiviral resistance can develop but it is important to allow for the fact that their experimental setup does not necessarily reflect what happens in either immunocompetent or immunocompromised individuals treated with antivirals.

  Response: *Thank you for addressing this. We agree, we do not think that low-frequency mutations have to be preexisting prior to selection (but it likely increases the adaptation potential). We softened our language **(line 91).***

- **Point 16:** 2. The authors propose that a 'quasispecies' assessment could be performed on patients with HSV-1 infection as a means of predicting the likelihood of antiviral resistance developing. This reads as significant over-interpretation, not least because they do not offer any evidence that such 'quasi-species' exist within the patients themselves, nor is it clear how this could realistically be performed. While the authors partially acknowledge the first part (lines 350-352), I would argue for minimizing the second part.

  Response: We have addressed this thoughtful comment throughout the manuscript. This concern was also raised by another reviewer. Please kindly refer to **Point 6** for a detailed explanation.

- **Point 17:** 3. The use of the term quasispecies is questionable as most standard definitions (in the context of viruses) would require the presence of large numbers of variant genomes that make up a population. However, while it is clear that a proportion of HSV-1 genomes in the sample are variant, by far the population is represented by a single dominant variant. I would argue that an alternative term should be used to describe this such as to prevent readers conflating this with the more standard definition that is usually applied to RNA viruses. Terms such as 'minority (genome) variants' have been proposed before within the herpesvirus field and would seem a better fit here.

  Response: We agree that the term quasi-species is controversial and have refrained from using it throughout the manuscript. Please kindly refer to **Point 1** of Reviewer 2 for a more detailed explanation.

- **Point 18:** Many numbers are written with a full stop rather than commas (e.g. 100.000 rather than 100,000)

  Response: Thank you, we fixed it at multiple occasions in the manuscript.

Reviewer #2:

- **Point 19:** Methods – Rescue of recombinant HSV-1 – Were the BAC-derived viruses plaque-purified after recovery from transfection into Vero cells? This should be stated yes/no, for

comparison to strain F, which was plaque-purified at the start. It may be useful to include this distinction in the results and/or figure legends, since it contributes an additional bottleneck to the experimental design (i.e., in addition to the bottleneck of low MOI 0.001).

<u>Response:</u> *Thank you for raising an important point which was not clarified by us properly. Initially, the two recombinant BAC-derived virus stocks (K17+ and K17+UL23(P84L)) were provided by collaboration partners and were not reconstituted by our group. The rescued viruses are described in a previous publication. Here, the authors passaged the rescued viruses multiple times to increase the viral titers (DOI: 10.1038/srep29903). Despite our best efforts, we were unable to reconstruct without doubt the passaging history of any of the used viruses in Figure 1. We previously mentioned this only in the discussion. Now we also state the passage history in the results section in* ***lines 107, 261-262.***

Furthermore, we have now clearly stated in the Methods that we did not produce the recombinant viruses for Figure 1/2 experiments ourselves ***(line 395-396).***

Notably, and this relates to the following points, only for the serial passaging experiment, we rescued the K17 wt, performed a plaque purification and used these stocks as the input for the passaging experiment (P0). We now state this in ***lines 266-288, 441-443*** *and* ***445-449*** *and the* ***legend of figure 5****.*

- **<u>Point 20:</u>** The methods (line 379) refer to the F-strain used here as being plaque-purified at the start. The initial isolate from Ejercito et al (citation 41) was not plaque-purified, and subsequent evolution studies have shown that starting from a bottlenecked population leads to a different evolutionary rate of change, than starting from a mixed population (PMC8389525, PMC7151645). This aspect of the experimental design should likely be mentioned in the Results and/or the Discussion.

  <u>Response:</u> *We have to sincerely apologize. We indeed stated that the F-Strain was plaque purified. However, this was also done by a collaborating laboratory that provided the initial stock from which we again grew our own virus stock (similar to* ***Point 19*** *regarding the BAC-derived viruses). Although the F-Strain was plaque-purified throughout its passaging history, this was not done directly prior to our experiments. We have corrected our statement accordingly* ***(line 395).***

- **<u>Point 21:</u>** Methods – serial virus passaging (lines 426-434). This section describes the serial passaging methods for the K17+_UL23(P84L) BAC-derived virus. As above, it would be useful to include the calculated PFU in the 10 ul starting population, and the estimated or known PFU in 10 ul transferred at later passages. In addition, a section should be added to the methods that describes the serial passaging details for the main experimental design (i.e., Figure 1A). That would enable a full comparison and/or contrast with this section.

  <u>Response:</u> *Thank you for raising this point as we did not clarify this properly. We initially picked three different plaques for the serial passaging experiments, cultured them individually for 48 h (P0) and then used 10 µl as the infection dose for P1 (please also refer to Point 19). The P0 titer was highly similar to the titer in the consecutive passages as titers reached similar levels in all cases after 48 h. The measured titers of each harvested passage are given in Supplementary Figure 8. With 10 µl, we transferred ~28,000 pfu per passage (calculated from the mean of log-*

*transformed values over all passages and replicates). We now also show the initial titer of P0 in **Supplementary Figure 8** and clarified the passaging experiment in the methods section including the mean titer **(line 449-451).***

*Both the seven-day growth kinetics (Fig.1a) and serial passaging experiment are already disclosed in the methods. We changed 'Growth kinetics' now to 'Viral growth in the presence or absence of ACV for seven days' in the Methods for clarification **(line 425).***

- **Point 22:** Multiple studies have shown that the HSV-1 strain F harbors sequence variations in its stock population. Strain F has been used in several experimental evolution series, either from an F-derived BAC (PMC11226790, PMC9888220) or an F-stock (PMC7151645). It would be useful to compare the low-frequency variants that are observed here, to those prior studies, to help infer whether the outcomes are deterministic or random.

  Response: *We thank the reviewer for his/her suggestion. At this point such a comparison is out of the scope of this study.*

- **Point 23:** The design of serial passaging experiments used here might also be called an experimental evolution setup – a term often used in RNA virus evolutionary studies. It may be helpful to the reader to introduce that concept here, and draw parallels to the rich history of RNA virus experimental evolution, and to related studies of HSV-1 and HSV-2.

  Response: *Thank you for the excellent suggestion. This also helps a lot to clearly separate the serial passaging from the seven days growth kinetics. We incorporated this term in **line 264 and line 444.***

- **Point 24:** Line 119 – The authors make a point that the titer in ACV-treated virus samples eventually reaches that of the mock-treated samples, but this occurs only because of resource limitation. The cell-resources in the mock-treated samples are likely exhausted days earlier than in the ACV-treated conditions (as evidenced by Figure 1 and Supp Fig 1 and 2). In addition, the titer of the mock-treated samples goes down by later days (see Supp Fig. 1), likely due to lysed cells and a drop in pH. This constraint on the interpretation of extended time-course data like these should be mentioned to give context to the ability of ACV-treated viruses to "catch up".

  Response: *We completely agree, the cytopathic effect was the limiting factor here as most cells were lysed after 72 h. We now mention these potential effects in **line 119-121**.*

- **Point 25:** Methods, lines 379-383. Was F-strain the only one plaque-purified, or were FR or FS as well? The level of starting variation seems similar across all three (see Supp Fig 6A) suggesting either that the plaque-purification wasn't much of a bottleneck, or that the methods should have specified that all three were treated similarly.

  Response: *This is an excellent observation and also relates to **Point 19/20** where we clarified this. None of the viruses used in the first experiments were plaque purified and had a documented passaging history. The only time plaque purification was applied was during the experimental evolution in Figure 5.*

- **<u>Point 26:</u>** It would be helpful to provide the context for the chosen levels of ACV (4um and 65um), e.g., in terms of past work or authors' hypotheses. Was there an assessment of Vero growth at the higher ACV dose, e.g., to ascertain lack of toxicity?

  <u>Response:</u> *We initially choose ACV concentrations to be within the range of the lower and upper plasma peak concentrations in patients who were treated intravenously with ACV, and to be well below the cytotoxic levels in cell culture. Published studies report steady-state plasma ACV concentrations ranging from the low micromolar level at trough to several micromolar at peak following intravenous dosing.*

  We did not perform a formal cytotoxicity assay, as ACV cytotoxicity in Vero and other mammalian cell lines has been assessed extensively in prior studies and occurs at concentrations substantially higher than those used here. Nevertheless, to confirm the absence of overt toxicity under our experimental conditions, we initially monitored uninfected Vero cells by light microscopy for seven days in the presence of mock treatment, 4 µM ACV, or 62 µM ACV. As expected, we observed no apparent cytotoxic effects at either concentration. Representative images of uninfected Vero cells at day 7 under these conditions are included in the following Figure 1, for review only.

  We have now provide context for both the dosing and cytotoxicity in the text citing relevant literature ***(lines 101-103).***

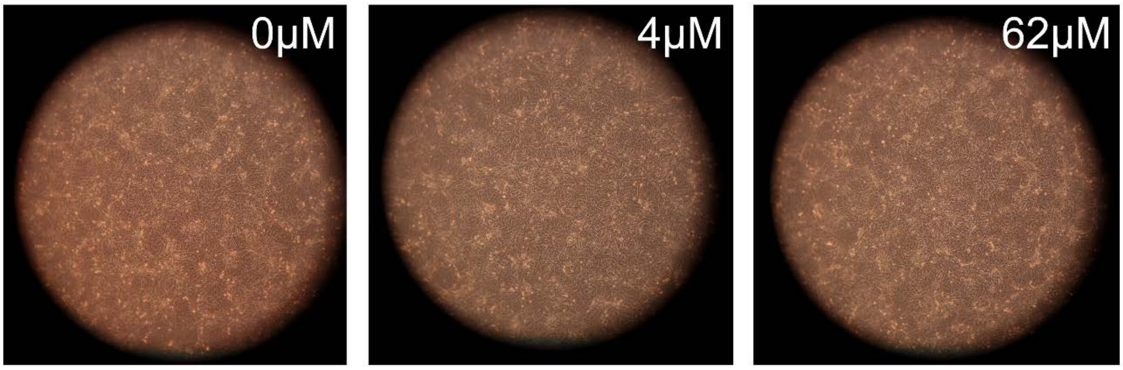

Figure 1. *ACV treated Vero cells for seven days with the indicated concentrations of ACV.*

Minor points for clarity:

- **<u>Point 27:</u>** Abstract & summary: Both of these short-form paragraphs include statements about long-term ACV exposure leading to resistance (lines 26, 49), without mentioning the important aspect that this occurs mainly in immunocompromised people. The main text is more clear (e.g., line70). Please mention this key clinical aspect in both the abstract and the summary, so as not to unintentionally mislead readers.

  <u>Response:</u> We fully agree with the reviewer. We now state that resistance is primarily an issue in immunocompromised patients in both the abstract and author summary **(line 26, 35, 46)**.

- **<u>Point 28:</u>** Abstract – What is meant by "error-borne"? Is this distinct from "error-prone"?

Response: *We thank the reviewer for noting this, rephrased the sentence and refrained from using error-borne in the updated manuscript.*

- **Point 29:** Line 30 – "Resulted" is not the right verb here, since growth kinetics and IC50 measurement did not cause or result in ACV resistance. Please correct.

  Response: *Thank you for the suggestion, we rephrased the sentence* ***(line 30).***

- **Point 30:** Lines 344-346 – This is not a new finding or proposal, and it has been stated in many primary research papers and many reviews already. Please include this context for the readers.

  Response: *We would like to thank the reviewer for pointing this out. It was not our intention to suggest a new mechanism, but to compare it with existing literature. We rephrased and provide context for the reader* ***(lines 366-368).***

- **Point 31:** ACV resuspension is not described in methods; please specify.

  Response: *ACV was resuspended in water into a stock solution of 110 mM used for the individual experiments. We now state this in Material and Methods* ***(line 415-416).***

- **Point 32:** Figure 1 – For clarity, please mention the use of biological triplicates in the figure legend, since it relates to the figure data and images.

  Response: *Thank you for the suggestion. We now state the use of 3 biological replicates clearly in the legend.*

- **Point 33:** Figure 1 and 2 – The circle sizes to indicate viral load are hard to distinguish sequentially (largest vs. smallest is easier). Could these be alternated hollow/solid or in some other way differentiated better for clarity?

  Response: *Thank you for the suggestion. The main reason for this "atypical" depiction of growth kinetics is that we wanted to draw the reader's attention to the 'time delay', establish a direct link between the IC_{50} values and each replicate, and present data on all viruses in a very condensed form. As the size of the circle is directly proportional to the viral titer, it is not possible to alternate between a hollow and a solid circle, since the measured viral titers are never exactly e.g. 10^5. The legend is therefore a point of reference which is common for these types of depictions. To address the issue and to show the data also as typical viral growth kinetics, we already included them in the supplements (both figures use exactly the same data – to avoid confusion we now explicitly state this in the figure description of Suppl. Fig 1 and 2).*

- **Point 34:** Line 726 – "K17+" should this read "K17+ passaged viruses"?

  Response: *Thank you for the suggestion, we rephrased the legend of supplementary figure 2.*

- **Point 35:** Supp Fig 6 - Legend states the authors' intention that "P-values are indicated for significant ANOVA results." However the figure shows many P-values that are not significantly different. To emphasize the places where significance is present, it would be better to remove the labeling of non-significant comparisons.

  Response: *Thank you for pointing this out. All p-values were shown for multiple testing results independent of their significance as multiple testing was only performed for significant ANOVA results. To clearly point out significant changes, we now only show p-values for p<=0.05. For consistency, this strategy was applied to all figures where we computed statistics.*

- **Point 36:** Line 735 – "synonymous variants/kb" should likely read "synonymous low-frequency variants/kb", to make clear to the reader that this figure is presenting low-frequency differences from the overall dominant population, not the overall genotype.

  Response: *We kindly disagree with this notion, as it would not be correct. Each viral sequence was constructed using de novo assembly, and the variants compared to this reference are given. The reference by itself is the dominant population. However, it is possible that, during remapping, mutations with a higher frequency are introduced (e.g. 0.3 would be included here as it is lower than the consensus threshold). So, while the term low-frequency would likely apply to the large majority of mutations, it's not correct in all cases.*

- **Point 37:** Figure 3C – When exposed to ACV (4 uM or 62 uM), there appear to be two mutations that get "fixed" (appear in all) replicates. These appear to be UL20 and UL44 or UL45 (unlabeled on left axis). Are these at the same site in each of these genes? If so, how would this arise in both treatments independently, but in all replicates of each treatment? If the sites are different but in the same gene, are these somehow conferring a selective advantage, such that they appear in all of these replicates?

  Response: *We appreciate the comment. All mutations in the treated settings are indeed the same mutations. They are present at a similar frequency in the parental virus and their frequency consistently increases in the treated viruses (see following figure). As we did not observe a clear pattern for the other viruses, we do not believe that UL20/UL44 mutations are directly involved in antiviral resistance or conferring any other replication advantage. We would rather speculate that these mutations are selected with the UL23 mutations, because they are part of the same parental subpopulation (due to the similar frequency of the parental virus and its consistent increase in frequency during drug treatment). We think that this is too speculative to be included in the manuscript. However, we have now included (see **Point 9**) a table showing the frequency with which UL23 and UL30 mutations over threshold are present in the respective samples and parental viruses.*

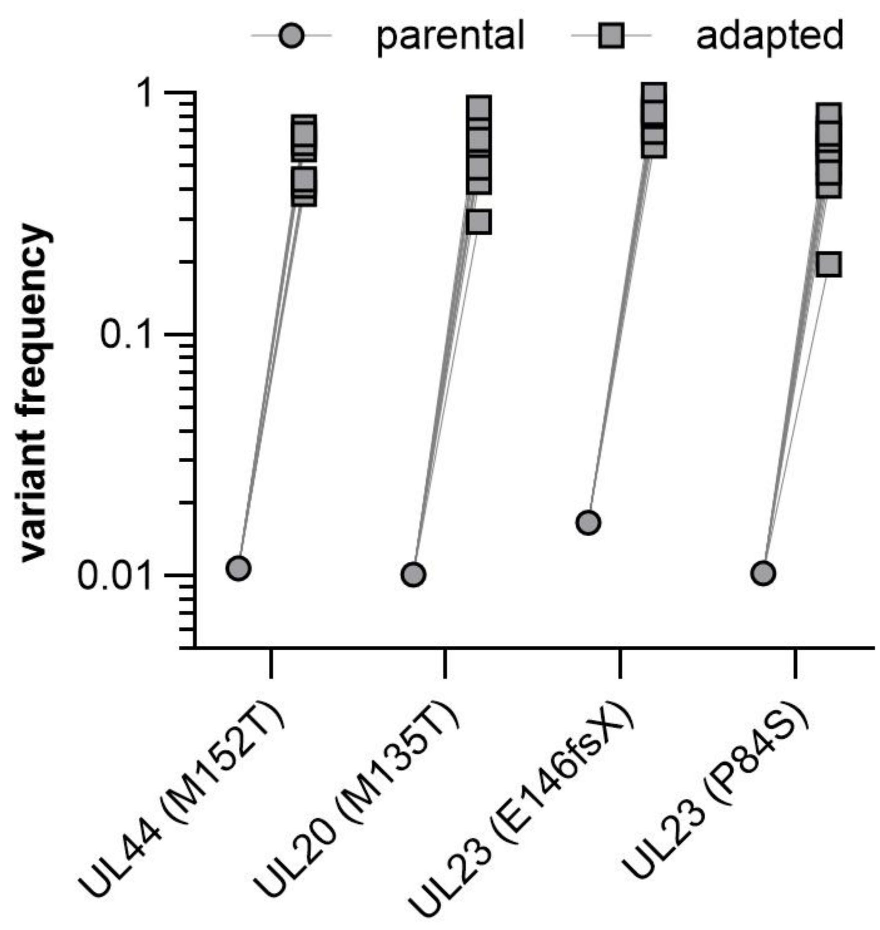

Figure 2. *Variant frequencies of UL44, UL20 and UL23 mutations of F-strain treated with 4 µM or 62 µM. Each dot represents the frequency of one replicate or parental virus.*

- **Point 38:** Figure 3A – the x-axis labeling on these panels is difficult to relate to. One can guess that it follows the order of Suppl. Table 1, but it would be easier if the labels were simply the same.

  Response: *Thank you for this suggestion. We changed the labelling and indicate this in the legend of Figure 3.*

- **Point 39:** Figure 3C, following on this, there are two columns (labels unclear; see above comment) that have many mutations – one in K17+_UL23P84L (2nd to last column of these) and one in the FR_sensitive (last column). Was there anything unusual about these two, such that their variance is so different than the others?

  Response: *We have rechecked the sequencing results and we do not have an obvious explanation why we have found more mutations in these cases above the cutoff. Moreover, we changed the labelling. This is now consistent with the previous figure and with the provided table for the coverage and genome recovery results.*

- **Point 40:** Figure 3C – left-axis labels are hard to follow across, and the labeling sometimes skips by more than one gene-interval. Were genes grouped for this analysis? If so, how? Can this be labeled more clearly?

  Response: *Thank you for spotting this! Each row represents a single gene. However, the matplotlib graphing library performed some automatic formatting of the y-axis, which we have corrected in the revised figure.*

- **Point 41:** Figure 5 – the extensive filtering here would likely not be necessary, if more stringent quality controls and cutoffs were applied to quantification of low-frequency variations. If the authors implement quality standards, panels C-D may not be necessary.

  Response: *Thank you for addressing this issue. We kindly refer to Point 13. The filtering step is merely descriptive and a way to analyse how the viral population changed during passaging. The filtering steps are not needed to identify the relevant (>5-fold increasing) mutations. We would like to emphasise here, that we not only exclude low frequency mutations with this filtering step but quite a lot of 'higher' frequency mutations. Therefore, more stringent filters, which exclude mutations at lower variant frequencies, will not exclude positions at higher frequencies that are excluded in this step.*

  As a reference, we include here the variant tracking in the absence of a filter.

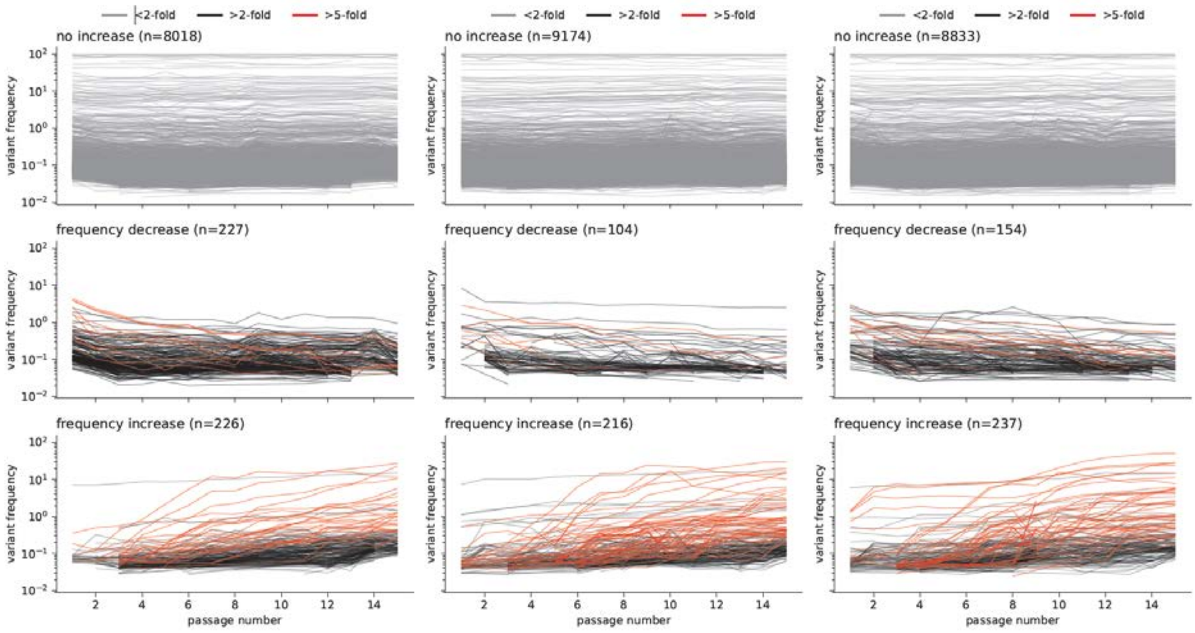

Figure 3. *Variant tracking in the absence of prior filtering. Same as Figure 5c but without the filtering step.*

- **Point 42:** Supplementary Figure 3 says "the coding sequences of the individual reference genes are plotted", but these do not appear to have been included. Please add these.

Response: *We apologize for this. It was an error from an earlier version of the figures which we forgot to delete. We decided not to show all the coding sequences as this would make the diagram too crowded and uninformative. We have deleted this sentence in the updated figure legend.*

- **Point 43:** Supplementary Figure 4 –The large number of non-synonymous variants differs from parallel peer studies of experimental evolution in HSV-1, suggesting that the inclusion of extremely low frequency variants (0.1 and below) skews these results. This should be re-computed with more rigorous criteria for what constitutes a low-frequency variant. Please also clarify if those genes missing bars are true "zero" values, or if these are genes that could not be analyzed in these strain(s) (e.g., due to sequencing gaps). This applies to UL11, US12, RL1, RL2, RS1 in various strains.

  Response: *We agree that these results differ from those of other studies. We do not think that the results are skewed per se as with increased sequencing depth more variants at lower frequencies are called (kindly refer to the next point and following figure). Differences are likely due to differences in variant calling cutoffs which relates to Point 2 of this rebuttal. We do not think that a re-computation with e.g. a variant cutoff of 0.1 would be beneficial. This graph was made to visualize that our analysis is consistent over different genes within one parental virus and is not intended as a comparison to other studies. In the absence of a widely accepted variant cutoff point that will exclude all non-true variants for HSV-1, we do not think that an exclusion of a subset of variants is beneficial.*
  The bars indicate zero values. We show the mean coverage in Supplementary Figure 4 as a proxy for comparable coverage over different genes. As this could also be due to over-proportional mapping in specific regions, while other regions are not mapped, we rechecked the per-base-coverages, which are consistent for all four genes (see following figure).

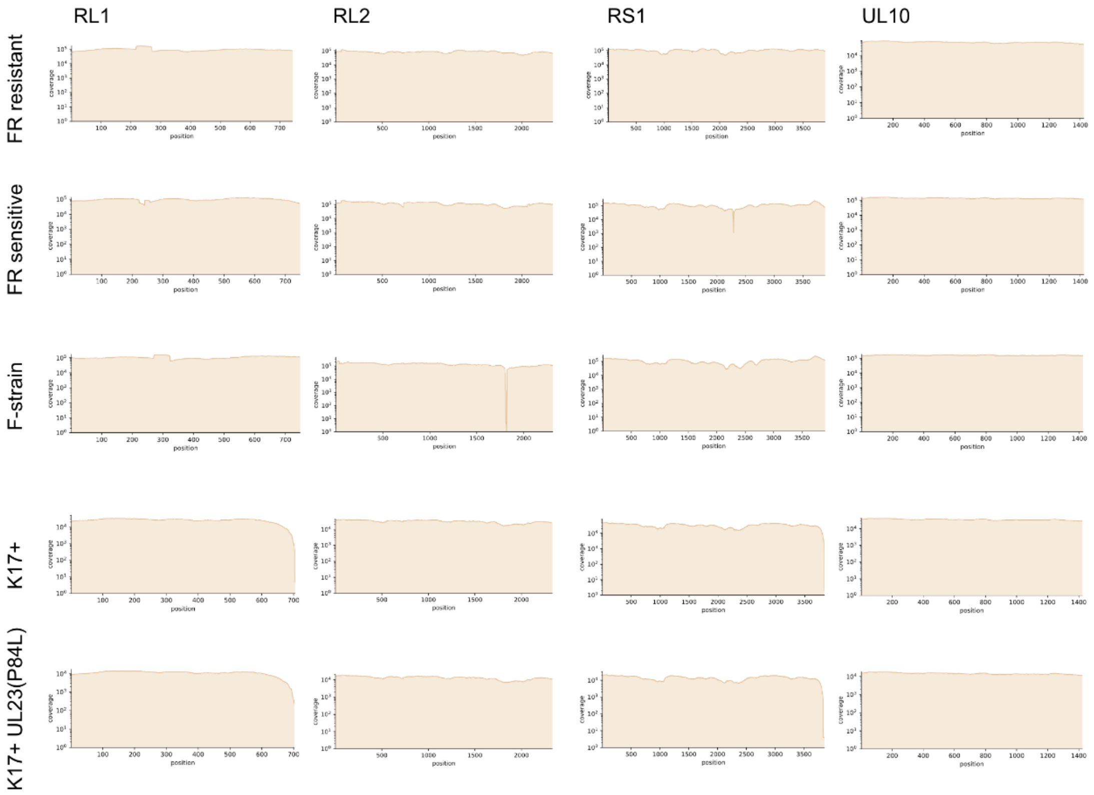

Figure 4. *Per base coverage plots for RL1, RL2, RS1 and UL10 of the five parental viruses.*

- <u>**Point 44:**</u> Supplementary Figure 5 – It would be insightful to plot the distribution of minor variant frequencies alongside these plots of the change in variant frequency, to give the reader a sense of the actual variant frequencies in each starting population.

  *<u>**Response:**</u> Thank you for this suggestion but we gently disagree that this is beneficial. Low-frequency mutations from the parental virus are only considered for the delta-variant frequency computation if they are also found in the mock treated samples (please kindly refer to Point 2). All mock treated samples have a lower sequencing depth by several logs and therefore identified mutations are higher in their frequency by default. Therefore, most low-frequency mutations of the parental virus do not influence the delta variant frequency (see **Supplementary Figure 6** for a detailed rationale for these analyses). Nevertheless, we have included the data here (see the following figure). However, we prefer not to show this in the manuscript, to avoid misinterpreting what we are actually analysing in this figure.*

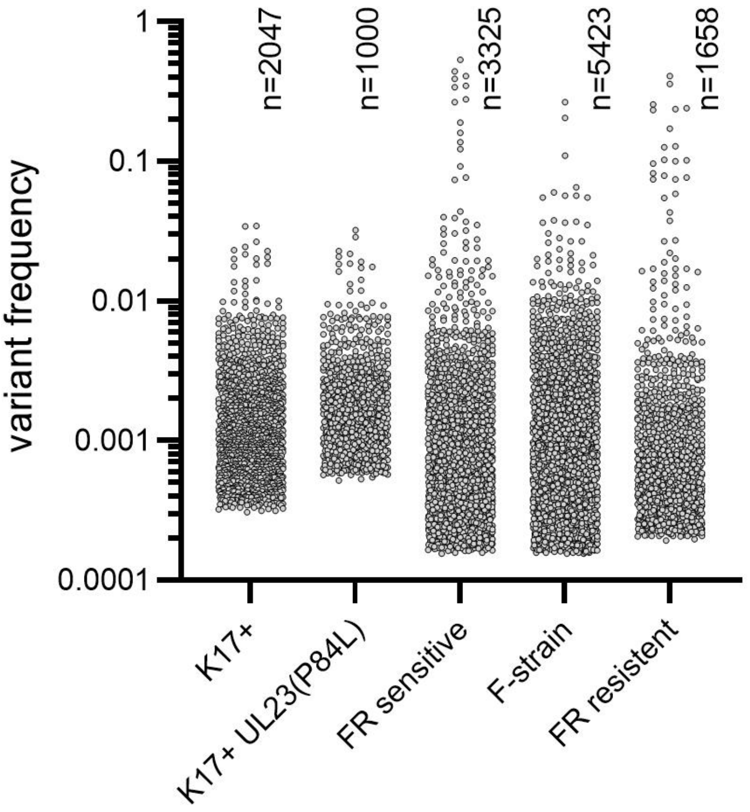

Figure 5. *Raw variant frequencies of all mutations in coding regions.*

Reviewer #3:

(No Response)
