# Supplementary material for: Low frequency variants can predetermine antiviral drug resistance development in herpes simplex virus type 1
Source: PLoS Pathog. 2026 Jun 8;22(6):e1014296. doi: 10.1371/journal.ppat.1014296 (PMC13262928; doi:10.1371/journal.ppat.1014296)
Supplement: Peer review history

# Peer review: Decision Letter 0

**DOI:** 10.1371/journal.ppat.1014296.r001

**Authors:** Anthony Nicola

31 Dec 2025

-->PPATHOGENS-D-25-02800

Viral quasi-species predetermine antiviral drug resistance development in herpes simplex virus type 1

PLOS Pathogens

Dear Dr. Fuchs,

Thank you for submitting your manuscript to PLOS Pathogens. After careful consideration, we feel that it has merit but does not fully meet PLOS Pathogens's publication criteria as it currently stands. Therefore, we invite you to submit a revised version of the manuscript that addresses the points raised during the review process.

Please submit your revised manuscript by Mar 01 2026 11:59PM. If you will need more time than this to complete your revisions, please reply to this message or contact the journal office at plospathogens@plos.org. When you're ready to submit your revision, log on to https://www.editorialmanager.com/ppathogens/ and select the 'Submissions Needing Revision' folder to locate your manuscript file.

Please include the following items when submitting your revised manuscript:

* A letter that responds to each point raised by the editor and reviewer(s). You should upload this letter as a separate file labeled 'Response to Reviewers'. This file does not need to include responses to any formatting updates and technical items listed in the 'Journal Requirements' section below.

* A marked-up copy of your manuscript that highlights changes made to the original version. You should upload this as a separate file labeled 'Revised Manuscript with Track Changes'.

* An unmarked version of your revised paper without tracked changes. You should upload this as a separate file labeled 'Manuscript'.

If you would like to make changes to your financial disclosure, competing interests statement, or data availability statement, please make these updates within the submission form at the time of resubmission. Guidelines for resubmitting your figure files are available below the reviewer comments at the end of this letter.

We look forward to receiving your revised manuscript.

Kind regards,

Anthony Nicola

Academic Editor

PLOS Pathogens

Donna Neumann

Section Editor

PLOS Pathogens

Sumita Bhaduri-McIntosh

Editor-in-Chief

PLOS Pathogens

orcid.org/0000-0003-2946-9497

Michael Malim

Editor-in-Chief

PLOS Pathogens

orcid.org/0000-0002-7699-2064

**Additional Editor Comments:**

Three experts have reviewed your manuscript and have provided detailed comments. One recommended minor revision, and two recommended major revision. Please address the comments and include a point-by-point response with the revised manuscript. Reviewers 1 and 2 take issue with the use of the term quasispecies, and I tend to agree with them. Thank you for submitting your work to PLOS Pathogens.

**Journal Requirements:**

1) We do not publish any copyright or trademark symbols that usually accompany proprietary names, eg ©,  ®, or TM  (e.g. next to drug or reagent names). Therefore please remove all instances of trademark/copyright symbols throughout the text, including:

- ® on page: 14 and 16.

2) Please upload all main figures as separate Figure files in .tif or .eps format. For more information about how to convert and format your figure files please see our guidelines:

https://journals.plos.org/plospathogens/s/figures

3) Some material included in your submission may be copyrighted. According to PLOSu2019s copyright policy, authors who use figures or other material (e.g., graphics, clipart, maps) from another author or copyright holder must demonstrate or obtain permission to publish this material under the Creative Commons Attribution 4.0 International (CC BY 4.0) License used by PLOS journals. Please closely review the details of PLOSu2019s copyright requirements here: PLOS Licenses and Copyright. If you need to request permissions from a copyright holder, you may use PLOS's Copyright Content Permission form.

Please respond directly to this email and provide any known details concerning your material's license terms and permissions required for reuse, even if you have not yet obtained copyright permissions or are unsure of your material's copyright compatibility. Once you have responded and addressed all other outstanding technical requirements, you may resubmit your manuscript within Editorial Manager.

Potential Copyright Issues:

- Figure 1A. Please confirm whether you drew the images / clip-art within the figure panels by hand. If you did not draw the images, please provide (a) a link to the source of the images or icons and their license / terms of use; or (b) written permission from the copyright holder to publish the images or icons under our CC BY 4.0 license. Alternatively, you may replace the images with open source alternatives. See these open source resources you may use to replace images / clip-art:

- https://commons.wikimedia.org

- https://openclipart.org/.

4) Please amend your detailed Financial Disclosure statement. This is published with the article. It must therefore be completed in full sentences and contain the exact wording you wish to be published.

State what role the funders took in the study. If the funders had no role in your study, please state: "The funders had no role in study design, data collection and analysis, decision to publish, or preparation of the manuscript.".

If you did not receive any funding for this study, please simply state: u201cThe authors received no specific funding for this work.u201d

**Reviewers' Comments:**

Reviewer's Responses to Questions

**Part I - Summary**

Please use this section to discuss strengths/weaknesses of study, novelty/significance, general execution and scholarship.

Reviewer #1: The manuscript by Jaki et al aims to investigate how phenotypic resistance to HSV-1 antivirals (primarily acyclovir) develop on a genomic level. Through a series of carefully designed experiments, they observe a low-level ‘quasispecies’ phenomenon within HSV-1 populations that provide a backbone for developing antiviral resistance. The manuscript is well written and the conclusions are generally supported by the data presented.

Reviewer #2: This manuscript by Jaki et al uses different starting stocks of herpes simplex virus 1 strains 17 and F, as the basis for an exploration of viral adaptation to aciclovir (ACV) exposure. HSV-1 strains 17 and F are classic experimental models for the study of HSV-1 (though here the authors rebrand strain F as a clinical isolate rather than a laboratory model strain). These strains are used either from the starting point of a BAC-clone (for strain 17) or a plaque-purified lab stock (strain F). In each case, the authors include “positive controls” of previously-described ACV-resistant variants of these strains for comparison. The viral stocks are then subjected to serial passaging (i.e., experimental evolution) in Vero cells either with or without ACV present. A variety of creative extensions of these passaging experiments are applied. The key findings of the paper are that a sizable number of low-frequency “mutations” (variants) are present in all of the HSV-1 stocks that they examine, and that these mutations can serve as the seeds for rapid viral adaptation to achieve ACV resistance. The paper includes a solid experimental design, good replication of results, and a clear progression through their findings. Issues in need of attention include the methods for quantifying these low-frequency variants (i.e., absent or unclear quality control on variant calling), and the choice of language and interpretation (e.g., use of quasi-species, clinical isolate terms). These and other more minor recommendations are detailed below.

Reviewer #3: In this manuscript, the authors analyzed the impact of ACV (acyclovir) selective pressure on different strains of herpes simplex virus 1 (HSV-1) in vitro. Following growth kinetics and IC50 evaluation, the authors concluded that development of ACV resistance occurred within a single passage. By using ultra-deep, non-targeted full-genome Illumina sequencing of the parental and ACV-adapted HSV-1 strains, ACV resistance mutations rapidly arose in the viral genes UL23 and UL30 and the authors claimed that they were already present in the parental ACV-naïve strains at extremely low variant frequencies. They hypothesized that low-variant frequencies represent an error-borne quasi-species swarm that develops during continued viral replication. They used a primary rescued recombinant K17+ strain and by continued passaging, an increased in the viral quasi-species pool was detected at low variant frequencies and allowed resistance development after 10 consecutive passages but not before. They deduced that viral quasispecies are a crucial prerequisite in the evolution of herpesviruses like HSV-1, enabling adaptation to selective pressures and that viral quasi-species assessment could allow prediction of a drug resistance potential. The authors have done a huge amount of work but some of the conclusions are well known (i.e., viral quasispecies are a crucial prerequisite in the evolution of herpesviruses) and others are quite controversial (i.e. rapid emergence of resistance after a single passage and the presence of ACV resistance mutations in the parental ACV-naïve strains at extremely low variant frequencies). Before this paper can be considered for publication, several points need to be clarified.

**********

**Part II – Major Issues: Key Experiments Required for Acceptance**

Please use this section to detail the key new experiments or modifications of existing experiments that should be absolutely required to validate study conclusions.

Generally, there should be no more than 3 such required experiments or major modifications for a "Major Revision" recommendation. If more than 3 experiments are necessary to validate the study conclusions, then you are encouraged to recommend "Reject".

Reviewer #1: (No Response)

Reviewer #2: - Use of the term “quasi-species” (in the title, abstract, summary, and main text) is not correct by definition, nor is the parallel helpful to the reader in the way it appears to be being used here. In virus evolution parlance, the term quasi-species is generally applied to RNA virus populations, where selection is postulated to act on a swarm of non-identical genomes that all carry mutations that could lead to lower fitness if they existed alone. In a viral quasi-species, the sum total of co-existing variants performs better than any single member, and could thus be maintained or constantly regenerated under evolutionary selection. Neither of these principles appear to apply to the observed data here, and nor are the relevant evolutionary aspects of quasi-species discussed in the manuscript or central to the results. Instead, the authors observed a very low number of HSV genomes that carry mutations, which co-exist with a very large percentage of non-mutant or intact genomes that dominate the viral population (e.g., most variants exist at frequencies <0.001). While the presence of the low-frequency variants is important, this viral population is not functioning as a quasi-species. The authors elsewhere use the terms “minority variants” (line 321), “low-variant frequencies” (line 339), and “low-frequency mutations” – any or all of which would work better throughout the manuscript (including title, abstract, summary, etc.).

- Quantification of low-frequency mutations – A key finding of the manuscript is its documentation of low-frequency mutations in the starting viral stocks, and its demonstration of how ACV-resistance mutations can be selected from this swarm or cloud of variation. The high allele frequencies achieved by the ACV-resistance mutations demonstrate that the overall findings are valid. However the quantitation of low-frequency variants lacks sufficient rigor. The methods mention use of the fastp package for quality control of Illumina sequence reads, but no parameters are stated. After read mapping, it is standard practice to apply criteria for the detection of low-frequency variants (e.g., PMCID: PMC10120596). These would typically include a minimum variant frequency (e.g. 1-2%) minimum coverage depth (e.g., 50-100X), bidirectional read support, and a minimum number of reads supporting the variant allele (e.g., 9 in PMC10120596). None of these appear to be applied in this study. The top row of Figure 5C illustrates the outcome of applying no filters, with most of the >5,000 variants in each panel having a frequency below 0.1 – a level that likely includes noise and are rarely included in peer studies. Without rigorous quality control or the use of unique molecular identifiers (UMIs) during library preparation, it is not clear that these extremely rare variants should be included. The focus on “change in variant frequency” in Figures 3-4 obscures the rarity of these variants. Technically, a variant that has a measured frequency of 0.1 and rises to a level of 0.5 has changed by 5% -- but may be entirely within the level of noise. Without rigorous quality control or UMIs, the statistical approaches used to pick an acceptable frequency cutoff for each experiment (Supp. Fig 1) fall short of the standards recommended for variant detection in herpesviruses (e.g., PMC10120596).

- Methods for sequence feature analysis – The methods for the analysis of sequence “regions that might cause problems” (line 224), and the related Supp. Fig. 6, are completely missing. While the idea of this sequence feature analysis is good, the chosen thresholds are generous and not explained. E.g., “homopolyX” (mono-nucleotide runs) are counted from as little as three of the same consecutive bases. Similar analyses often start at a level of 4. Other short repeats (e.g., di-, tri-nucleotides ) are counted from a simple dyad on up. These generous settings inflate the number of instances detected, and should be justified if used. The right-hand side of each analysis panel displays the breakdown of these findings, and highlights the large number in each category that are at the “bare minimum” level of meeting the detection criteria. The left-hand panel uses the aggregate numbers to establish significance, and there is a concern that the large number of “bare minimum” loci may be inflating significance. This analysis would be more impactful if run with a more rigorous requirement for what constitutes each sequence feature.

- Figure 1 / experimental design – it would be useful to include an estimate of the starting viral population in addition to the MOI (i.e., calculated from volume of actual dish(es) used for infection). This is relevant because at an MOI of 0.001, the total PFU input dictates the potential rarity of introducing very rare mutants from the starting population. By stating the PFU, it helps the reader understand the size of the starting population, and provides a more straightforward comparison with other experimental-evolution studies.

- HSV-1 strain F was isolated from a patient lesion originally, but it has since been propagated in many laboratories for many passages, and it is widely considered to be a reference or laboratory strain (e.g., PMCID: PMC516408, PMC7764767). For the HSV field, the term “clinical isolate” usually refers to recent patient isolates of a known and/or low passage number. This is not the case for strain F, and the text should be adjusted accordingly (e.g., lines 108, 111, & throughout).

Reviewer #3: A major issue regarding this study is the choice of cells if one wants to extrapolate the results to the clinic. Vero cells are a continuous (immortalized) lineage of African green monkey kidney epithelial cells. They are aneuploid and are interferon-deficient, which may impact the time to select drug-resistant viruses. Thus, selection of drug-resistant viruses should be more relevant in primary human cells (either fibroblasts or epithelial cells) where HSV-1 easily grows. Therefore, the conclusions the authors drawn from their study should be much more cautious.

Line 151, “We were able to reconstruct highly covered viral genomes with mean coverage of up 152 to 100.000-fold (Suppl. Fig. 3).” There are quite some differences in coverage along the HSV-1 genome, with some regions showing very low coverage. What is the coverage along the genes of interest UL23 and UL30? Suppl. Fig 4 shows the mean coverage per gene but not the variations in coverage along the UL23 and UL30 genes. Different individual positions of interest could exhibit deviating coverages.

What is the False Discovery Rate (FDR) cut-off? Strict threshold should be used to ensure high confidence in called variants (1% to 5%).

The results should be presented in a more clear way to facilitate the reading of the manuscript. For instance, which mutations did emerge following ACV pressure (Fig. 1)? Where these mutations detected in the parental virus? Figure 4 shows the mutations conferring resistance in UL23 and UL30 already present in the viral stocks but it’s not specified in which parental virus there were present and under which condition they were selected. Which ACV-resistance mutations were acquired by the each parental virus?

Fig. 1b. There are quite some differences in sensitivity to ACV among the HSV-1 viruses that are sensitive to ACV. The K17+ (BAC-derived) has a substantial lower IC50 value than the F-strain and FR_sensitve isolate. This will certainly impact the fold-resistance.

It is very hard to understand why ACV resistance mutations would develop and increase over consecutive passaging in the absence of ACV pressure. The authors should demonstrate that these data are reproducible that there are no errors related to detection of minor viral variants. If ACV resistance would emerge so easily, it should occur much more frequently in the clinic than what is reported.

Fig 5 is very difficult to follow. Is it correct that most of the mutations detected when passaging the viruses are not detected at #15? Which viral variants emerged as the dominant ones and are related to ACV resistance?

The authors should be careful with the statement ‘viral quasi-species assessment could allow prediction of a drug resistance potential’. There is a general concern in the scientific community on the clinical significance of low-prevalence mutations (detected by NGS at a prevalence between 5 and 15%). It is crucial to analyze patients with low-prevalence mutations and how they respond to treatment over time to define the potential significance of minor variant populations. Besides, not all mutations detected at low frequency will emerge as the domini ant variant since they may have a high fitness cost, which will be influenced by the drug selective pressure. The authors should precautious in giving recommendations based only in in vitro studies.

**********

**Part III – Minor Issues: Editorial and Data Presentation Modifications**

Please use this section for editorial suggestions as well as relatively minor modifications of existing data that would enhance clarity.

Reviewer #1: My only significant critiques of the work are as follows:

1. I do not think the experiments definitively prove that the development of resistance requires the presence of pre-existing low frequency mutations (lines 95-97). This can mostly be addressed by simply softening the language used to state that their data supports this as one mechanism by which antiviral resistance can develop but it is important to allow for the fact that their experimental setup does not necessarily reflect what happens in either immunocompetent or immunocompromised individuals treated with antivirals.

2. The authors propose that a ‘quasispecies’ assessment could be performed on patients with HSV-1 infection as a means of predicting the likelihood of antiviral resistance developing. This reads as significant over-interpretation, not least because they do not offer any evidence that such ‘quasi-species’ exist within the patients themselves, nor is it clear how this could realistically be performed. While the authors partially acknowledge the first part (lines 350-352), I would argue for minimizing the second part.

3. The use of the term quasispecies is questionable as most standard definitions (in the context of viruses) would require the presence of large numbers of variant genomes that make up a population. However, while it is clear that a proportion of HSV-1 genomes in the sample are variant, by far the population is represented by a single dominant variant. I would argue that an alternative term should be used to describe this such as to prevent readers conflating this with the more standard definition that is usually applied to RNA viruses. Terms such as ‘minority (genome) variants’ have been proposed before within the herpesvirus field and would seem a better fit here.

4. Many numbers are written with a full stop rather than commas (e.g. 100.000 rather than 100,000)

Reviewer #2: - Methods – Rescue of recombinant HSV-1 – Were the BAC-derived viruses plaque-purified after recovery from transfection into Vero cells? This should be stated yes/no, for comparison to strain F, which was plaque-purified at the start. It may be useful to include this distinction in the results and/or figure legends, since it contributes an additional bottleneck to the experimental design (i.e., in addition to the bottleneck of low MOI 0.001).

- The methods (line 379) refer to the F-strain used here as being plaque-purified at the start. The initial isolate from Ejercito et al (citation 41) was not plaque-purified, and subsequent evolution studies have shown that starting from a bottlenecked population leads to a different evolutionary rate of change, than starting from a mixed population (PMC8389525, PMC7151645). This aspect of the experimental design should likely be mentioned in the Results and/or the Discussion.

- Methods – serial virus passaging (lines 426-434). This section describes the serial passaging methods for the K17+_UL23(P84L) BAC-derived virus. As above, it would be useful to include the calculated PFU in the 10 ul starting population, and the estimated or known PFU in 10 ul transferred at later passages. In addition, a section should be added to the methods that describes the serial passaging details for the main experimental design (i.e., Figure 1A). That would enable a full comparison and/or contrast with this section.

- Multiple studies have shown that the HSV-1 strain F harbors sequence variations in its stock population. Strain F has been used in several experimental evolution series, either from an F-derived BAC (PMC11226790, PMC9888220) or an F-stock (PMC7151645). It would be useful to compare the low-frequency variants that are observed here, to those prior studies, to help infer whether the outcomes are deterministic or random.

- The design of serial passaging experiments used here might also be called an experimental evolution setup – a term often used in RNA virus evolutionary studies. It may be helpful to the reader to introduce that concept here, and draw parallels to the rich history of RNA virus experimental evolution, and to related studies of HSV-1 and HSV-2.

- Line 119 – The authors make a point that the titer in ACV-treated virus samples eventually reaches that of the mock-treated samples, but this occurs only because of resource limitation. The cell-resources in the mock-treated samples are likely exhausted days earlier than in the ACV-treated conditions (as evidenced by Figure 1 and Supp Fig 1 and 2). In addition, the titer of the mock-treated samples goes down by later days (see Supp Fig. 1), likely due to lysed cells and a drop in pH. This constraint on the interpretation of extended time-course data like these should be mentioned to give context to the ability of ACV-treated viruses to “catch up”.

- Methods, lines 379-383. Was F-strain the only one plaque-purified, or were FR or FS as well? The level of starting variation seems similar across all three (see Supp Fig 6A) suggesting either that the plaque-purification wasn’t much of a bottleneck, or that the methods should have specified that all three were treated similarly.

- It would be helpful to provide the context for the chosen levels of ACV (4um and 65um), e.g., in terms of past work or authors’ hypotheses. Was there an assessment of Vero growth at the higher ACV dose, e.g., to ascertain lack of toxicity?

Minor points for clarity:

- Abstract & summary: Both of these short-form paragraphs include statements about long-term ACV exposure leading to resistance (lines 26, 49), without mentioning the important aspect that this occurs mainly in immunocompromised people. The main text is more clear (e.g., line70). Please mention this key clinical aspect in both the abstract and the summary, so as not to unintentionally mislead readers.

- Abstract – What is meant by “error-borne”? Is this distinct from “error-prone”?

- Line 30 – “Resulted” is not the right verb here, since growth kinetics and IC50 measurement did not cause or result in ACV resistance. Please correct.

- Lines 344-346 – This is not a new finding or proposal, and it has been stated in many primary research papers and many reviews already. Please include this context for the readers.

- ACV resuspension is not described in methods; please specify.

- Figure 1 – For clarity, please mention the use of biological triplicates in the figure legend, since it relates to the figure data and images.

- Figure 1 and 2 – The circle sizes to indicate viral load are hard to distinguish sequentially (largest vs. smallest is easier). Could these be alternated hollow/solid or in some other way differentiated better for clarity?

- Line 726 – “K17+” should this read “K17+ passaged viruses”?

- Supp Fig 6 - Legend states the authors’ intention that “P-values are indicated for significant ANOVA results.” However the figure shows many P-values that are not significantly different. To emphasize the places where significance is present, it would be better to remove the labeling of non-significant comparisons.

- Line 735 – “synonymous variants/kb” should likely read “synonymous low-frequency variants/kb”, to make clear to the reader that this figure is presenting low-frequency differences from the overall dominant population, not the overall genotype.

- Figure 3C – When exposed to ACV (4 uM or 62 uM), there appear to be two mutations that get “fixed” (appear in all) replicates. These appear to be UL20 and UL44 or UL45 (unlabeled on left axis). Are these at the same site in each of these genes? If so, how would this arise in both treatments independently, but in all replicates of each treatment? If the sites are different but in the same gene, are these somehow conferring a selective advantage, such that they appear in all of these replicates?

- Figure 3A – the x-axis labeling on these panels is difficult to relate to. One can guess that it follows the order of Suppl. Table 1, but it would be easier if the labels were simply the same.

- Figure 3C, following on this, there are two columns (labels unclear; see above comment) that have many mutations – one in K17+_UL23P84L (2nd to last column of these) and one in the FR_sensitive (last column). Was there anything unusual about these two, such that their variance is so different than the others?

- Figure 3C – left-axis labels are hard to follow across, and the labeling sometimes skips by more than one gene-interval. Were genes grouped for this analysis? If so, how? Can this be labeled more clearly?

- Figure 5 – the extensive filtering here would likely not be necessary, if more stringent quality controls and cutoffs were applied to quantification of low-frequency variations. If the authors implement quality standards, panels C-D may not be necessary.

- Supplementary Figure 3 says “the coding sequences of the individual reference genes are plotted”, but these do not appear to have been included. Please add these.

- Supplementary Figure 4 –The large number of non-synonymous variants differs from parallel peer studies of experimental evolution in HSV-1, suggesting that the inclusion of extremely low frequency variants (0.1 and below) skews these results. This should be re-computed with more rigorous criteria for what constitutes a low-frequency variant. Please also clarify if those genes missing bars are true “zero” values, or if these are genes that could not be analyzed in these strain(s) (e.g., due to sequencing gaps). This applies to UL11, US12, RL1, RL2, RS1 in various strains.

- Supplementary Figure 5 – It would be insightful to plot the distribution of minor variant frequencies alongside these plots of the change in variant frequency, to give the reader a sense of the actual variant frequencies in each starting population.

Reviewer #3: (No Response)

**********

PLOS authors have the option to publish the peer review history of their article (what does this mean?). If published, this will include your full peer review and any attached files.

If you choose “no”, your identity will remain anonymous but your review may still be made public.

**Do you want your identity to be public for this peer review?**  For information about this choice, including consent withdrawal, please see our Privacy Policy.

Reviewer #1: No

Reviewer #2: No

Reviewer #3: No

[NOTE: If reviewer comments were submitted as an attachment file, they will be attached to this email and accessible via the submission site. Please log into your account, locate the manuscript record, and check for the action link "View Attachments". If this link does not appear, there are no attachment files.]

**Figure resubmission:**

-->While revising your submission, we strongly recommend that you use PLOS’s NAAS tool (https://ngplosjournals.pagemajik.ai/artanalysis) to test your figure files. NAAS can convert your figure files to the TIFF file type and meet basic requirements (such as print size, resolution), or provide you with a report on issues that do not meet our requirements and that NAAS cannot fix.

After uploading your figures to PLOS’s NAAS tool - https://ngplosjournals.pagemajik.ai/artanalysis, NAAS will process the files provided and display the results in the "Uploaded Files" section of the page as the processing is complete. If the uploaded figures meet our requirements (or NAAS is able to fix the files to meet our requirements), the figure will be marked as "fixed" above. If NAAS is unable to fix the files, a red "failed" label will appear above. When NAAS has confirmed that the figure files meet our requirements, please download the file via the download option, and include these NAAS processed figure files when submitting your revised manuscript.

**Reproducibility:**

To enhance the reproducibility of your results, we recommend that authors of applicable studies deposit laboratory protocols in protocols.io, where a protocol can be assigned its own identifier (DOI) such that it can be cited independently in the future. Additionally, PLOS ONE offers an option to publish peer-reviewed clinical study protocols. Read more information on sharing protocols at https://plos.org/protocols?utm_medium=editorial-email&utm_source=authorletters&utm_campaign=protocols

# Author comment: Author response to Decision Letter 1

**DOI:** 10.1371/journal.ppat.1014296.r002

1 Apr 2026

Attachment Submitted filename: Response to Reviewers.pdf

# Peer review: Decision Letter 1

**DOI:** 10.1371/journal.ppat.1014296.r003

**Authors:** Anthony Nicola

26 May 2026

Dear Dr Fuchs,

We are pleased to inform you that your manuscript 'Low frequency variants can predetermine antiviral drug resistance development in herpes simplex virus type 1' has been provisionally accepted for publication in PLOS Pathogens.

Before your manuscript can be formally accepted you will need to complete some formatting changes, which you will receive in a follow up email. A member of our team will be in touch with a set of requests.

Please also make the remaining minor corrections recommended by Reviewer 2, and deposit the genomes to public archives to enable reuse.

Please note that your manuscript will not be scheduled for publication until you have made the required changes, so a swift response is appreciated.

IMPORTANT: The editorial review process is now complete. PLOS will only permit corrections to spelling, formatting or significant scientific errors from this point onwards. Requests for major changes, or any which affect the scientific understanding of your work, will cause delays to the publication date of your manuscript.

Should you, your institution's press office or the journal office choose to press release your paper, you will automatically be opted out of early publication. We ask that you notify us now if you or your institution is planning to press release the article. All press must be co-ordinated with PLOS.

Thank you again for supporting Open Access publishing; we are looking forward to publishing your work in PLOS Pathogens.

Best regards,

Anthony Nicola

Academic Editor

PLOS Pathogens

Donna Neumann

Section Editor

PLOS Pathogens

Sumita Bhaduri-McIntosh

Editor-in-Chief

PLOS Pathogens

orcid.org/0000-0003-2946-9497

Michael Malim

Editor-in-Chief

PLOS Pathogens

orcid.org/0000-0002-7699-2064

***********************************************************

Please make the remaining minor corrections recommended by Reviewer 2, and deposit the genomes to public archives to enable reuse.

Reviewer Comments (if any, and for reference):

Reviewer's Responses to Questions

**Part I - Summary**

Please use this section to discuss strengths/weaknesses of study, novelty/significance, general execution and scholarship.

Reviewer #1: The authors have addressed all of my concerns

Reviewer #2: The authors have responded with care to the extensive feedback of all three reviewers. The "toning down" of the language (e.g. around ACV resistance prediction in humans, & quasi-species) provides a more balanced presentation of the data. The addition of Supplementary Figure 5 -- to explain their chosen route of detecting minor variants -- is helpful. Only a few minor suggestions are included below; no re-review needed.

**********

**Part II – Major Issues: Key Experiments Required for Acceptance**

Please use this section to detail the key new experiments or modifications of existing experiments that should be absolutely required to validate study conclusions.

Generally, there should be no more than 3 such required experiments or major modifications for a "Major Revision" recommendation. If more than 3 experiments are necessary to validate the study conclusions, then you are encouraged to recommend "Reject".

Reviewer #1: (No Response)

Reviewer #2: (No Response)

**********

**Part III – Minor Issues: Editorial and Data Presentation Modifications**

Please use this section for editorial suggestions as well as relatively minor modifications of existing data that would enhance clarity.

Reviewer #1: (No Response)

Reviewer #2: Likely due to the rearrangements of several figures between versions 1 and 2, there are some minor details to correct. Figure 5 legend refers to a panel e that appears to actually be panel d.

Legend for Supplementary Table 2 is missing.

"Supplementary Data" appears to be another table; not sure why this is not just called Supplementary Table 3. It would also be helpful to add a line to legend to explain what the criteria was for the "detected mutations" in the parental viruses. The methods and text do not really address variant detection in the parentals, except to say that a complete absence of a variant was scored as "0". So by corollary, perhaps a single read in a parental strain would get it into this list? It would be good to simply state this.

**********

PLOS authors have the option to publish the peer review history of their article (what does this mean?). If published, this will include your full peer review and any attached files.

If you choose “no”, your identity will remain anonymous but your review may still be made public.

**Do you want your identity to be public for this peer review?**  For information about this choice, including consent withdrawal, please see our Privacy Policy.

Reviewer #1: No

Reviewer #2: No

# Editor report: Acceptance letter

**DOI:** 10.1371/journal.ppat.1014296.r004

**Authors:** Anthony Nicola

Dear Dr Fuchs,

We are delighted to inform you that your manuscript, "Low frequency variants can predetermine antiviral drug resistance development in herpes simplex virus type 1," has been formally accepted for publication in PLOS Pathogens.

We have now passed your article onto the PLOS Production Department who will complete the rest of the pre-publication process. All authors will receive a confirmation email upon publication.

The corresponding author will soon be receiving a typeset proof for review, to ensure errors have not been introduced during production. Please review the PDF proof of your manuscript carefully, as this is the last chance to correct any scientific or type-setting errors. Please note that major changes, or those which affect the scientific understanding of the work, will likely cause delays to the publication date of your manuscript. Note: Proofs for Front Matter articles (Pearls, Reviews, Opinions, etc...) are generated on a different schedule and may not be made available as quickly.

Soon after your final files are uploaded, the early version of your manuscript, if you opted to have an early version of your article, will be published online. The date of the early version will be your article's publication date. The final article will be published to the same URL, and all versions of the paper will be accessible to readers.

For Research Articles, you will receive an invoice from PLOS for your publication fee after your manuscript has reached the completed accept phase. If you receive an email requesting payment before acceptance or for any other service, this may be a phishing scheme. Learn how to identify phishing emails and protect your accounts at https://explore.plos.org/phishing.

Thank you again for supporting open-access publishing; we are looking forward to publishing your work in PLOS Pathogens.

Best regards,

Sumita Bhaduri-McIntosh

Editor-in-Chief

PLOS Pathogens

orcid.org/0000-0003-2946-9497

Michael Malim

Editor-in-Chief

PLOS Pathogens

orcid.org/0000-0002-7699-2064
